# Trends and Outcomes of TAVI and SAVR in Cancer and Noncancer Patients: A Nationwide Analysis

**DOI:** 10.1016/j.jacadv.2022.100167

**Published:** 2023-01-27

**Authors:** Waqas Ullah, Nishanth Thalambedu, Salman Zahid, Hamza Zahid Ullah Muhammadzai, Harigopal Sandhyavenu, Arnav Kumar, M. Chadi Alraies, Alec Vishnevsky, Nicholas J. Ruggiero, Mamas A. Mamas, Michael P. Savage, David L. Fischman

**Affiliations:** a Department of Cardiology, Thomas Jefferson University Hospitals, Philadelphia, Pennsylvania, USA; b Department of Cardiology, Abington Jefferson Health, Abington, Pennsylvania, USA; c Department of Cardiology, Rochester General Hospitals, Rochester, New York, USA; d Department of Cardiology, Weiss Memorial Hospital, Chicago, Illinois, USA; e Department of Cardiology, Brigham and Women’s Hospital Heart & Vascular Center, Boston, Massachusetts, USA; f Department of Cardiology, Detroit Medical Center, Detroit, Michigan, USA; g Department of Cardiology, Keele University, Keele, United Kingdom

**Keywords:** cancer, severe aortic stenosis, surgical aortic valve replacement, transcatheter aortic valve implantation

## Abstract

**Background:**

Patients with severe aortic stenosis and cancer are often denied surgical aortic valve replacement (SAVR) due to a prohibitive risk of perioperative mortality.

**Objectives:**

The purpose of this study was to determine the safety of transcatheter aortic valve implantation (TAVI) in patients with severe aortic stenosis and cancer.

**Methods:**

The Nationwide Inpatient Sample database (2002-2018) was used to study the outcomes of TAVI vs SAVR in patients with active or prior history of prostate, lung, colorectal, breast, and renal cancer. A propensity score-matched analysis to calculate adjusted odds ratios (aORs) for major adverse cardiovascular events (MACEs) and its components.

**Results:**

A total of 1,505,995 crude population and a subset of 345,413 noncancer and 33,565 cancer patients were selected on propensity score-matched analysis. The yearly trend showed a steep increase in the utilization of TAVI. Compared with SAVR, TAVI had a lower risk of in-hospital mortality in prostate cancer, while there was no difference among other cancer types. Patients with lung (aOR: 0.65; 95% CI: 0.43-0.97) and prostate cancer (aOR: 0.79; 95% CI: 0.66-0.96) had lower, while colorectal cancer (aOR: 1.43; 95% CI: 1.08-1.90) had higher odds of MACE with TAVI. The incidence of major bleeding was lower with TAVI (except for lung cancer), while the risk of stroke was similar (except for colorectal cancer) between TAVI and SAVR.

**Conclusions:**

TAVI in patients with prostate, breast, lung, and renal cancer appears to be a reasonable alternative to SAVR with lower or similar risks of mortality and MACE.

With early diagnosis and continuous advancement in cancer therapies, there has been a significant improvement in the life expectancy of oncology patients.^1^ This has paradoxically increased the prevalence of cardiovascular diseases in cancer-surviving adults. A growing body of literature has demonstrated a mechanistic overlap between cancer and heart diseases.^2^ Cardiovascular disease manifesting after cancer may be due to the shared risk factors profile, adverse effects of cancer therapies, and severe inflammatory state associated with malignancies. Specifically, the extension of potentially cardiotoxic chemotherapies to elderly populations with preexisting aortic stenosis (AS), and radiotherapy-induced damage to the aortic valve has resulted in the simultaneous presence of both severe AS and cancer. Recent reports show that the prevalence of malignancy in patients with severe AS is >26%.^3^ The improved survival following cancer treatment, coupled with the toxic impacts of cancer therapies on AS have made the latter an important determinant of morbidity and mortality in this patient cohort.^4^ Currently, there has been no consensus on the appropriate management of these patients who have severe AS and a history of cancer.

Surgical aortic valve replacement (SAVR) is limited due to increased perioperative mortality while the benefits of transcatheter aortic valve implantation (TAVI) are uncertain as patients with advanced cancers and those with an estimated life expectancy of fewer than 2 years were excluded from the landmark TAVI trials.^5^, ^6^, ^7^, ^8^, ^9^, ^10^ Hence, we sought to determine the relative merits of TAVI vs SAVR in patients with the most common cancers.

## Methods

### Data source

Nationwide Inpatient Sample (NIS) is the largest United States payer database of hospitalized patients that is publicly available through the Agency for Healthcare Research and Quality. It is managed and monitored by the Healthcare Cost and Utilization Project. It contains de-identified in-hospital data of weighted discharges for more than 15 million hospitalizations per year. Due to the anonymized nature of data, NIS is exempted from the approval of the institutional review board.

### Study design and population

The current study included all adult patients (>18 years of age) with a diagnosis of AS undergoing aortic valve replacement (AVR) between 2002 and 2018. The included sample was divided into 2 groups: patients undergoing TAVI vs SAVR. All comparisons were stratified based on the prior history of or active presence of one of the 5 major cancer types (breast, prostate, kidney, colorectal cancer (CRC), and lung). The standard International Classification of Disease, Clinical Modifications codes were used to identify different cancers, baseline comorbidities, and outcomes of interest (other than mortality). The detailed list of International Classification of Disease-10 codes is provided in Supplemental Table 1. Minors (age <18 years) and patients undergoing open-heart surgery for reasons other than isolated SAVR such as concomitant atrial and ventricular septal defect repair, tricuspid, pulmonic, or mitral valve replacement were excluded from the analysis.

### Study outcomes

The primary end point was a composite of stroke and in-hospital mortality termed as major adverse cardiovascular events (MACEs). Secondary end points consisted of individual components of MACE, major bleeding, postprocedural bleeding, and the need for permanent pacemaker (PPM) implantation. A detailed explanation of outcomes is given in Supplemental Table 2.

### Statistical analysis

**Figure 1:**
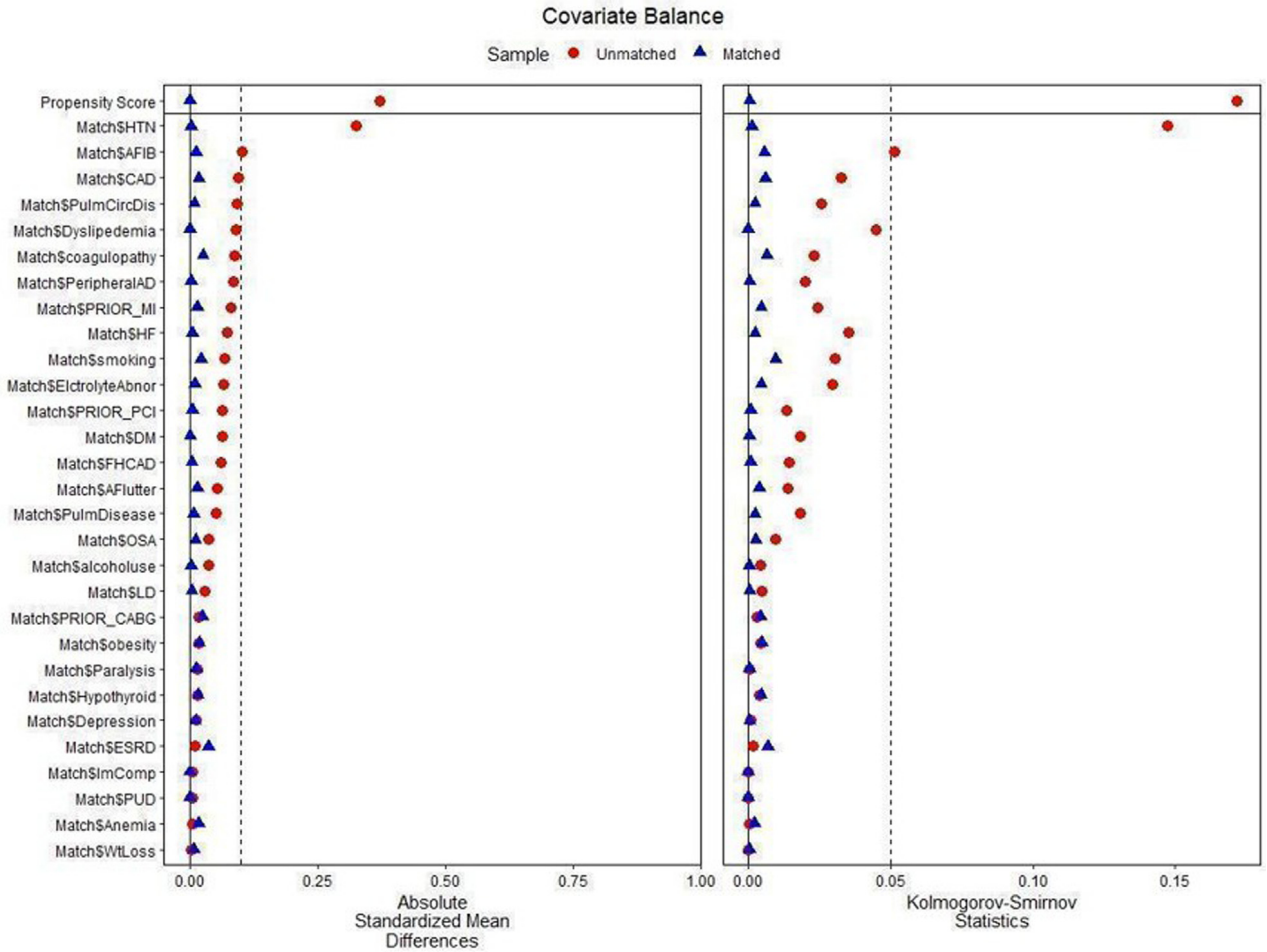
**Propensity Matched Analysis Showing the Standardized Mean Differences of Major Comorbidities Showing No Deviation Beyond the Allowable Threshold (0.1 SMD and 0.05 KSS)** AFIB = atrial fibrillation; CABG = coronary artery bypass graft; CAD = coronary artery disease; DM = diabetes mellitus; ESRD = end-stage renal disease; HF = heart failure; HTN = hypertension; KSS = Kolmogorov-Smirnov Statistics; MI = myocardial infarction; OSA = obstructive sleep apnea; PCI = percutaneous coronary intervention; PUD = peptic ulcer disease; SMD = standardized mean difference.

NIS discharge weights were used to obtain national estimates. Baseline comorbidities were compared using descriptive statistics. A chi-squared test was used to compare the percentages of categorical variables. The Cochran-Mantel Haenszel test was used to compute unadjusted odds ratios (ORs) for crude data. To compute adjusted odds ratios (aORs), a 2-step approach was adopted for each subgroup (no-cancer, CRC, prostate, lung, kidney, and breast); dealing with the missing values and generating a matched population having balanced baseline characteristics. The frequency of missing values was first calculated, and then Little’s MCAR (missing completely at random) test was used to screen for patterns of missing data. A significant value (*P* < 0.05) indicated systematic and a nonsignificant value (*P* > 0.05) represented randomly missing data. Trimming and Winsorizing methods were performed to exclude cases with <1% of missing data, while expectation-maximization was adopted to account for >1% randomly missing values. For each cancer group and noncancer patients, a stepwise multivariate propensity score matching (PSM) was performed using a 1:many near neighbor strategy without replacement of the matched cohort. The maximum tolerated standardized mean difference and Kolmogorov-Smirnov Statistics were set at a caliper of 0.1 and 0.05, respectively (Supplemental Figures 1 and 2). For PSM, more than 30 variables were balanced between the 2 comparison arms (TAVI vs SAVR) for each prespecified subgroup (Figure 1, Supplemental Table 3). The complete list of matching variables is given in Supplemental Table 1. A sensitivity analysis based on the sequential exclusion of potential covariates such as males, females, older adults, and those with nonactive and metastatic cancer was also performed to assess its impact on pooled outcomes. Using a multivariate interaction analysis, the intergroup estimates of major outcomes for TAVI vs SAVR groups were also calculated across males vs females and younger vs older populations. Linear regression analysis was used to assess the annual trends of the utilization of procedures. The trend analysis of annual percentage change (APC) for the overall cohort was also performed using the joinpoint regression method. The calculated effect sizes were analyzed using point estimates with its 95% CI and a type I error of *P* ≤ 0.05 was chosen as a cutoff for statistical significance. All analyses were performed using SPSS v24 (IBM Corp), R 3.2, and STATA v16 (StataCorp).

## Results

### Selection of cases

On the initial search, 38,349,112 weighted hospitalizations of aortic valve disease were identified. Of these, 1,505,995 patients underwent isolated AVR. Among these, 1,373,817 had no history of cancer, while 132,177 had a prior history of cancer or active cancer. From the latter, 104,003 patients with the most common cancer types were selected. Of these, 14,593 patients had active cancer and 4,412 had metastatic cancer (Supplemental Table 4). A weighted sample of 345,413 noncancer and 33,565 most common cancer propensity-matched populations were selected for adjusted analysis (Supplemental Figure 3). The detailed frequency of major cancer types undergoing TAVI vs SAVR is given in Table 1.

**Table 1:** Sample Size of Unmatched and Propensity-matched Populations Across No Cancer and Different Types of Cancer Undergoing TAVI vs SAVR

	Crude				Propensity			
	SAVR	Percentage	TAVI	Percentage	SAVR	Percentage	TAVI	Percentage
No cancer	1,012,968	93.00%	360,849	86.50%	171,923	91.50%	175,275	91.70%
Colorectal cancer	8,544	0.80%	7,408	1.80%	3,745	2.00%	4,255	2.20%
Kidney cancer	2,787	0.30%	2,500	0.60%	877	0.50%	890	0.50%
Lung cancer	2,901	0.30%	4,369	1.00%	1,273	0.70%	1,480	0.80%
Prostate cancer	22,675	2.10%	17,809	4.30%	6,476	3.40%	6,544	3.40%
Breast cancer	19,197	1.80%	15,814	3.80%	6,174	3.30%	6,294	3.30%

SAVR = surgical aortic valve replacement; TAVI = transcatheter aortic valve implantation.

### Baseline characteristics

**Figure 2:**
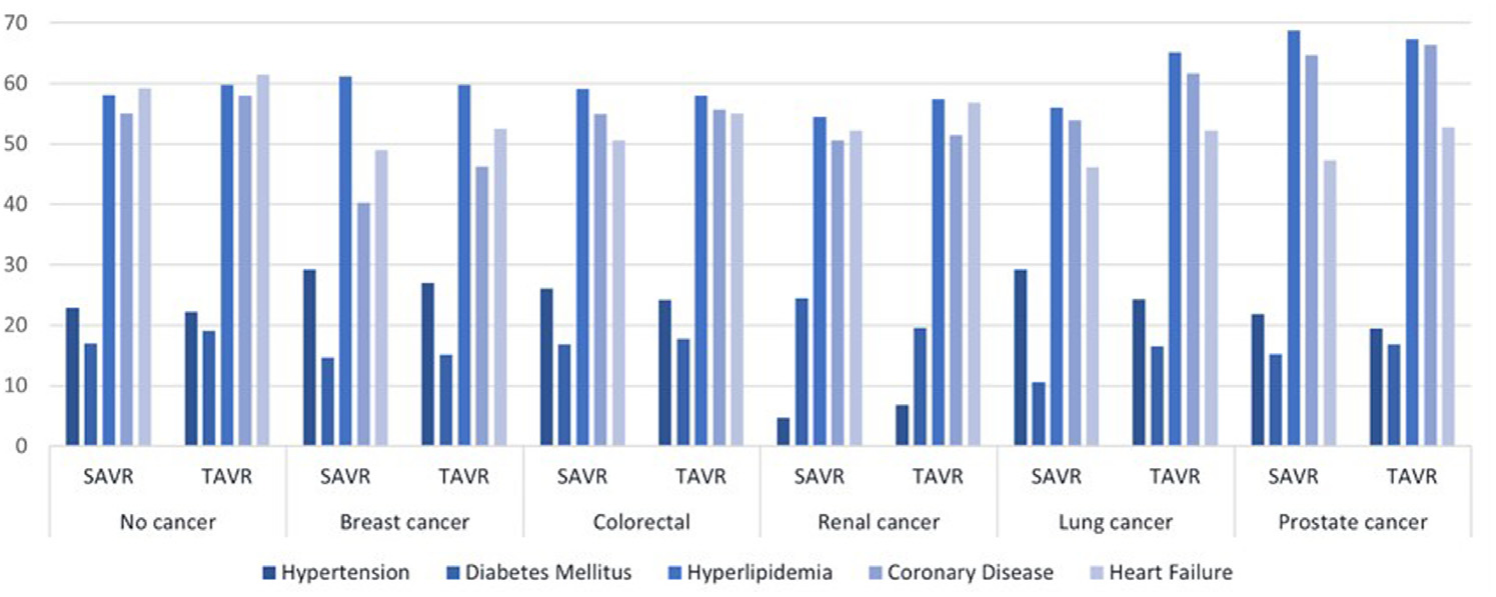
**Baseline Comorbidities Among Patients With No Cancer and Different Types of Cancers Undergoing SAVR vs TAVI on Propensity-Matched Analysis** SAVR = surgical aortic valve replacement; TAVI = transcatheter aortic valve implantation.

Detailed baseline comorbidities for the overall population on crude unadjusted analysis are presented in Supplemental Table 5. The mean age of patients undergoing TAVI was significantly higher than those having SAVR in all cancer groups. The majority of patients (>70%) in both groups were Caucasian. Patients undergoing TAVI had a significantly higher burden of traditional cardiovascular comorbidities irrespective of the type of cancer. More than 80% of the procedures were nonemergent and elective over the weekdays. Using PSM, the baseline covariates were evenly distributed in the matched groups (TAVI vs SAVR) across all cancer types. (Figure 2, Supplemental Figure 4, Tables 2 and 3).

**Table 2:** Baseline Crude Demographics of Patients Without Cancer and With Cancer

	No		Breast		Colorectal		Renal		Lung		Prostate	
	SAVR	TAVI	SAVR	TAVI	SAVR	TAVI	SAVR	TAVI	SAVR	TAVI	SAVR	TAVI
Sex												
Male	54.3	53.6	1.3	0.8	57.5	53.2	72.2	68.2	58.3	59.1	100	100
Female	45.7	46.4	98.7	99.2	42.5	46.8	27.8	31.8	41.7	40.9	0	0
Race												
White	79.6	79.8	84.8	83.4	81.8	86.2	82.5	79.7	87.1	85.2	82.4	80.3
Black	4	4.5	2.5	4	4.8	3.4	5.4	8.1	4	3.5	4.6	4.3
Hispanic	5.5	4.7	2.9	4.3	4.4	3.3	4.8	0	0	2.6	3.4	4
Asian	1.2	1.3	0.9	1.2	1.7	1.1	0	0	0	0.9	0.4	0.8
Native	0.4	0.3	1	0	1	0	0	0	0.9	0	0.2	0.2
Other	2.5	2.8	2.4	2.3	3	1.9	1.4	2.7	2.6	0.9	1.4	3
Unknown	6.7	6.6	5.5	4.8	3.3	4	5.9	9.5	5.4	7	7.7	7.6
Admission												
Nonelective	26.2	21.1	17.6	16.1	21.1	12.3	24.6	18.9	19	16.5	16.3	18.9
Elective	73.8	78.9	82.4	83.9	78.9	87.7	75.4	81.1	81	83.5	83.7	81.1
Payer (insurance)												
Medicare	83.9	86.5	88.6	88.9	90.9	88.8	77.5	82.4	88.9	83.5	88	89.3
Medicaid	1.9	2	0.5	1.5	1.3	0.7	3.4	2.7	1.3	0.9	0.8	0.3
Private	12.3	9.3	9.8	8.6	7	9	19.1	13.5	8.1	13.9	9.4	7.3
Self-pay	0.8	0.6	0.4	0.3	0.7	0.4	0	1.4	0	0.9	0.5	0.5
No charge	0.1	0	0	0	0	0	0	0	0	0	0	0
Other	1.1	1.6	0.7	0.7	0.2	1.1	0	0	1.7	0.9	1.3	2.6
Bed size												
Small	6.3	5.1	4.6	4.5	5.8	8.5	0	0	7.9	4.3	5.5	4.6
Medium	18.9	17.8	21.6	18.9	15.7	15.2	19.1	13	14.6	23.4	20.2	16
Large	74.8	77.2	73.8	76.6	78.5	76.3	80.9	87	77.5	72.3	74.3	79.4
Teaching status												
Rural	2.3	0.7	1.8	0.4	2.4	0	1.6	0	3.4	2.1	1.7	0.5
Urban, NT	27.5	9.3	26	8.5	22.9	10.4	17.2	4.3	22.7	6.4	26.5	11.9
Urban, T	70.2	89.9	72.2	91.1	74.6	89.6	81.2	95.7	73.8	91.5	71.8	87.6
Region												
Northeast	31.3	42.5	35.2	43.5	44.7	43.5	22.1	30.4	36.2	38.3	32.4	45.2
Midwest	21.3	21.8	18.2	22.2	13.2	20.6	25.9	21.7	24.1	23.4	20.6	21.9
South	26.5	23.6	23	22.6	22.3	26.4	25.2	26.1	24.1	25.5	24.1	21.5
West	20.8	12	23.6	11.8	19.8	9.4	26.8	21.7	15.7	12.8	22.9	11.4
Location												
Central	22.1	23.8	20.8	27	24.2	32.2	15.9	16.7	24.4	20.6	20	23.4
Fringe	25.8	25.9	27.3	29.4	30	26.1	46.6	40.2	29.3	25	29.9	29.2
Metro >250K-999K	22.1	22	26.9	21.5	18.8	19.6	12.5	27.5	35.4	26.5	20.8	21.4
Metro 50K-249K	10.7	10.4	10.8	7.9	12.1	6.7	11.4	3.9	2.4	14.7	15.1	12.7
Micropolitan	10.8	9.9	6.7	9.6	4.8	8.6	9.1	7.8	4.9	5.9	8	10
None	8.5	8.1	7.6	4.8	10.1	6.7	4.5	3.9	3.7	7.4	6.3	3.4

Values are %.

ACH = acute care health; NT = nonteaching; SAVR = surgical aortic valve replacement; TAVI = transcatheter aortic valve implantation.

**Table 3:** Propensity Matched Baseline Comorbidities of Patients Underwent TAVI and SAVR Without Cancer and With Different Cancers

	No Cancer		Breast Cancer		Colorectal		Renal Cancer		Lung Cancer		Prostate Cancer	
	SAVR	TAVI	SAVR	TAVI	SAVR	TAVI	SAVR	TAVI	SAVR	TAVI	SAVR	TAVI
Hypertension	22.9	22.2	29.2	27	26.1	24.2	4.7	6.8	29.2	24.3	21.8	19.5
Diabetes mellitus	17	19.1	14.6	15.1	16.8	17.8	24.5	19.6	10.6	16.5	15.2	16.8
Hyperlipidemia	58.1	59.8	61.2	59.8	59.1	58	54.5	57.4	56	65.2	68.8	67.3
Coronary disease	55	58	40.3	46.2	54.9	55.7	50.6	51.4	53.9	61.7	64.7	66.4
Smoking	26.6	27.4	22.8	23.3	32.4	34.2	38.8	39.9	48.3	48.7	38.3	41.9
Atrial fibrillation	42	42.1	40.9	39.1	41.2	44.2	38.5	42.6	40.6	45.2	43.9	43.5
Atrial flutter	5.4	5.2	4.2	3.6	4.8	3.7	7.5	4.7	5.7	5.2	5.4	4.7
Heart failure	59.2	61.5	49	52.5	50.6	55	52.2	56.8	46.1	52.2	47.3	52.8
Prior PCI	5.3	6	5.5	5.8	7.5	7.1	4.8	2.7	6.9	7	7.5	7
Prior CABG	11.1	13.1	4.3	5.7	11.7	14.9	8.6	8.1	12.7	10.4	15.8	19.3
Prior MI	7.8	9	5.5	8.1	6.7	6.7	5.1	8.1	9.9	10.4	7.8	9.2
Family history of CAD	4.8	5.2	7	6.2	6	6.3	7.5	6.8	5.2	8.7	5.4	5.5
Pulmonary disease	17.6	19.5	13.9	15.9	15.8	15.7	19.1	24.3	46.3	48.7	15	15.2
Pulmonary circulation disease	8.8	8.8	7.8	6.3	5.7	6.3	4.7	6.8	4.6	5.2	5.2	4.4
OSA	10.5	11	7.5	9	8.7	5.9	15	15.5	8.3	13	12	12
Anemia	3.1	3.5	3.2	3.3	2.9	2.2	2.7	4.1	2.2	6.1	3.1	3.6
Coagulopathy	2.8	2.5	2.2	1.8	2.4	1.5	1.4	1.4	2.5	1.7	2.1	1.7
PAD	8	8.4	6.2	6.6	7.8	8.2	6.2	6.8	8.5	12.2	5.6	6.3
Liver disease	2.5	2.5	1.4	1.8	1.9	1.9	0	0	0	0.9	0.8	1.1
Drug abuse	2	1.6	1.8	1.3	1.3	1.1	0	0	0.9	0.9	0.3	0.6
ESRD	4.6	4.9	1.5	1.8	3.2	3.7	13.6	10.8	1.7	0.9	2.6	2.4
Hypothyroid	14.8	15.7	26.5	25.3	17.9	17.1	13	16.2	17.9	13.9	8.1	9.8
Obesity	14.3	14.8	13.6	12.6	11.2	8.6	21.8	16.9	6.2	10.4	9.8	10.8

Values are %.

CABG = coronary artery bypass graft; CAD = coronary artery disease; ESRD = end-stage renal disease; MI = myocardial infarction; OSA = obstructive sleep apnea; PAD = peripheral arterial disease; PCI = percutaneous coronary intervention; SAVR = surgical aortic valve replacement; TAVI = transcatheter aortic valve implantation.

### Temporal trend analysis

**Figure 3:**
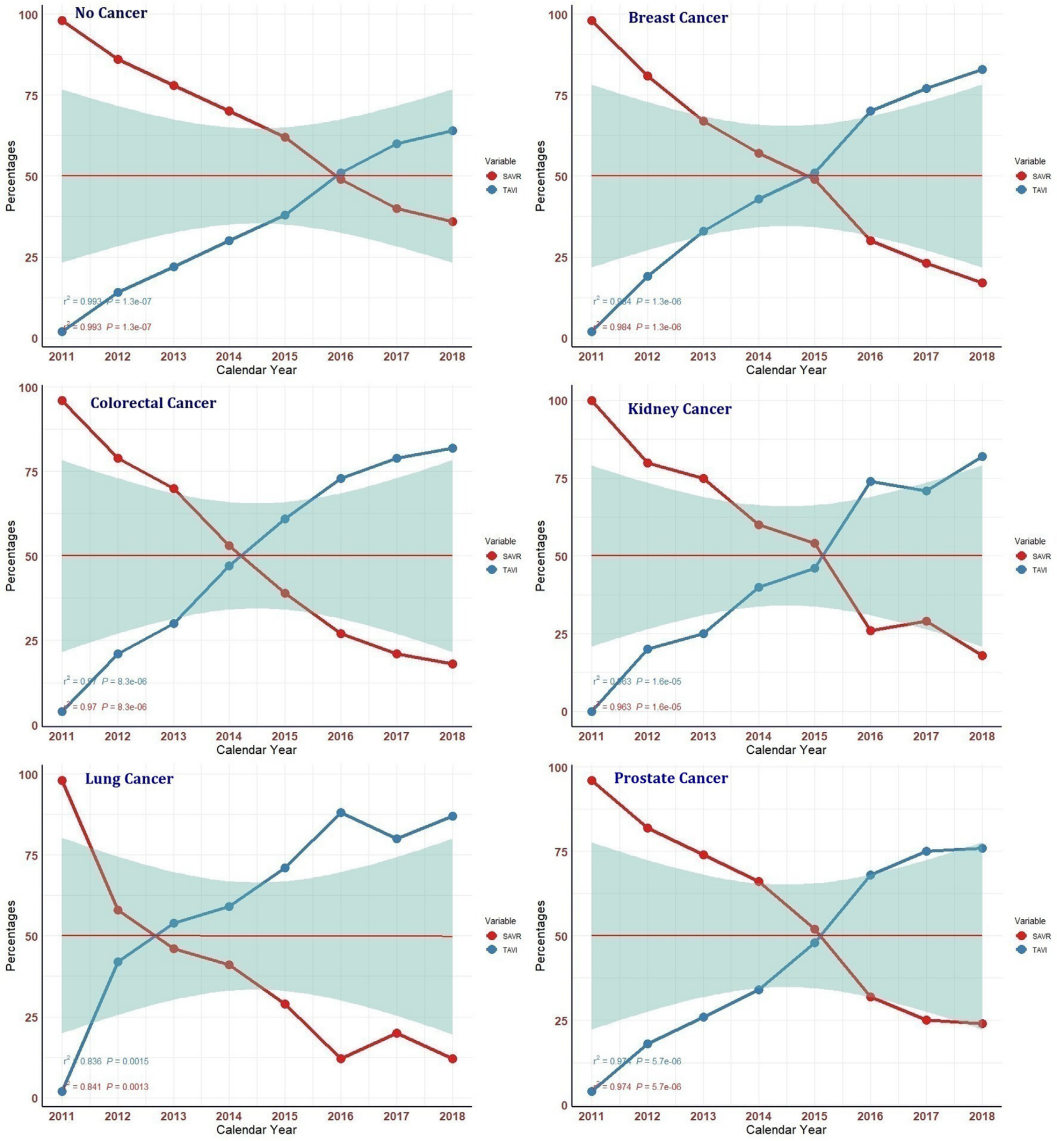
**Yearly Trend of SAVR and TAVI Procedures Performed for Patients With No Cancer, Breast, Colorectal, Lung, Prostate, and Renal Cancer** SAVR = surgical aortic valve replacement; TAVI = transcatheter aortic valve implantation.

The yearly trend showed a steep increase in the utilization of TAVI from 2011 to 2018 irrespective of the type of cancer (Supplemental Figure 5). By the year 2015, the proportion of TAVI procedures among all cancer patients had surpassed the corresponding proportion of SAVR procedures. TAVI in lung cancer observed this transition relatively earlier during 2012 (Supplemental Tables 6 and 7, Figure 3). There was a decline in the utilization of SAVR, (APC: −0.48%; 95% CI: −1.1% to 0.2%), and a significant increase in the use of TAVI (APC: 4.79%; 95% CI: 1.5%-11.5%) during the study period (2011-2018). Regarding the major outcomes, there was a decreasing trend observed in the percentage of MACE over the years in all cancer types. The risk of major bleeding and stroke was variable, while the need for PPM implantation remained significantly higher in TAVI across all timepoints regardless of the history and type of cancer (Supplemental Tables 8 and 9).

### Crude unadjusted analysis

On crude unadjusted analysis, the odds of MACE, mortality, and other procedure-related complications (stroke and major bleeding) were significantly lower with TAVI in patients with no history of cancer and those having prostate cancer. Patients with breast cancer had a higher, while those with lung and colorectal cancer had a similar risk of in-hospital mortality with TAVI compared with SAVR. The need for PPM remained significantly higher among TAVI groups irrespective of the type of malignancy. The detailed estimates of all unadjusted outcomes are given in Supplemental Tables 10 to 15.

### Propensity matched analysis

#### Pooled analysis of noncancer patients

**Figure 4:**
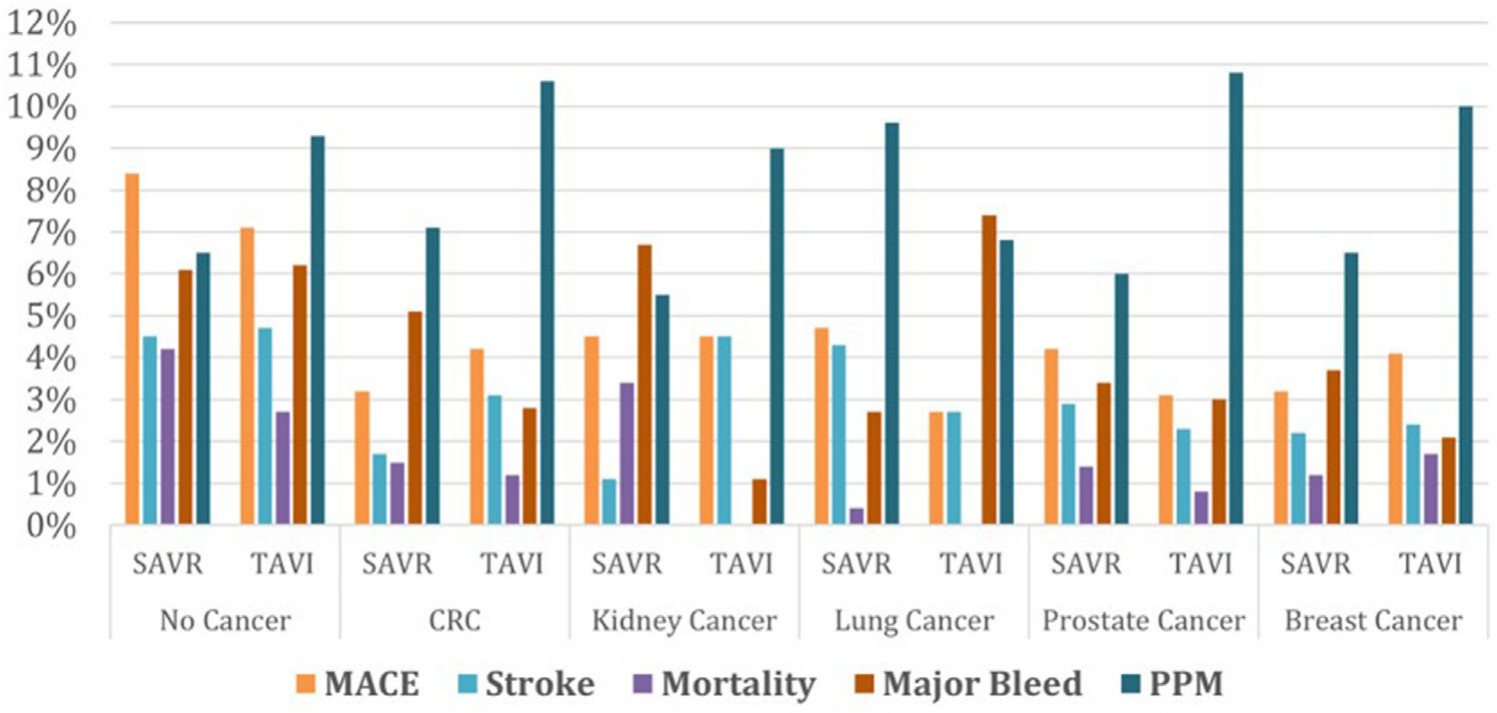
**Proportion of MACE, Stroke, Mortality, Major Bleed, and PPM Implantation Among Patients With No Cancer and Different Types of Cancers Undergoing SAVR vs TAVI on Propensity-Matched Analysis** CRC = colorectal cancer; MACE = major adverse cardiovascular events; PPM = permanent pacemaker; SAVR = surgical aortic valve replacement; TAVI = transcatheter aortic valve implantation.

Among patients with no history of cancer, the adjusted odds for MACE (aOR: 0.84; 95% CI: 0.82-0.87) and in-hospital mortality (aOR: 0.63; 95% CI: 0.60-0.65) were significantly lower, while the rates of stroke (aOR: 1.06; 95% CI: 1.02-1.09) and the need for PPM implantation (aOR: 1.46; 95% CI; 1.42-1.49) were higher with TAVI compared with SAVR (Table 4, Supplemental Table 10, Figure 4).

**Table 4:** Propensity Matched Outcomes Between TAVI and SAVR Among No Cancer and Different Cancer Patients

	No Cancer	Breast	Lung	CRC	Renal	Prostate
MACE	0.84 (0.82-0.87)	1.20 (0.99-1.45)	0.65 (0.43-0.97)	1.43 (1.08-1.90)	1.36(0.84-2.2)	0.79 (0.66-0.96)
Mortality	0.63 (0.60-0.65)	1.33 (0.98-1.80)	0.17 (0.02-1.4)	0.58 (0.33-1.01)	0.25(0.28-2.2)	0.65 (0.46-0.91)
Stroke	1.06 (1.02-1.09)	1.03 (0.81-1.32)	0.71 (0.47-1.07)	2.01 (1.43-2.84)	4.2(0.87-19)	0.82 (0.66-1.03)
PP bleed	0.48 (0.46-0.51)	0.44 (0.32-0.62)	---	0.11 (0.06-0.22)	---	0.34 (0.25-0.47)
Major bleed	1.02 (0.99-1.04)	0.63 (0.51-0.79)	3.08 (2.07-4.59)	0.66 (0.52-0.83)	0.11 (0.01-0.88)	0.85 (0.69-0.99)
PPM	1.46 (1.42-1.49)	1.60 (1.40-1.83)	0.62 (0.45-0.84)	1.67 (1.37-2.03)	3.76 (1.21-11.71)	2.02 (1.77-2.30)

Values are OR (95% CI).

AKI = acute kidney injury; CS = cardiogenic shock; HD = hemodialysis; IABP = intra-aortic balloon pump; MACE = major adverse cardiovascular events; PP = postprocedural; PPM = permanent pacemaker; SAVR = surgical aortic valve replacement; TAVI = transcatheter aortic valve implantation.

#### Pooled analysis of cancer patients

**Figure 5:**
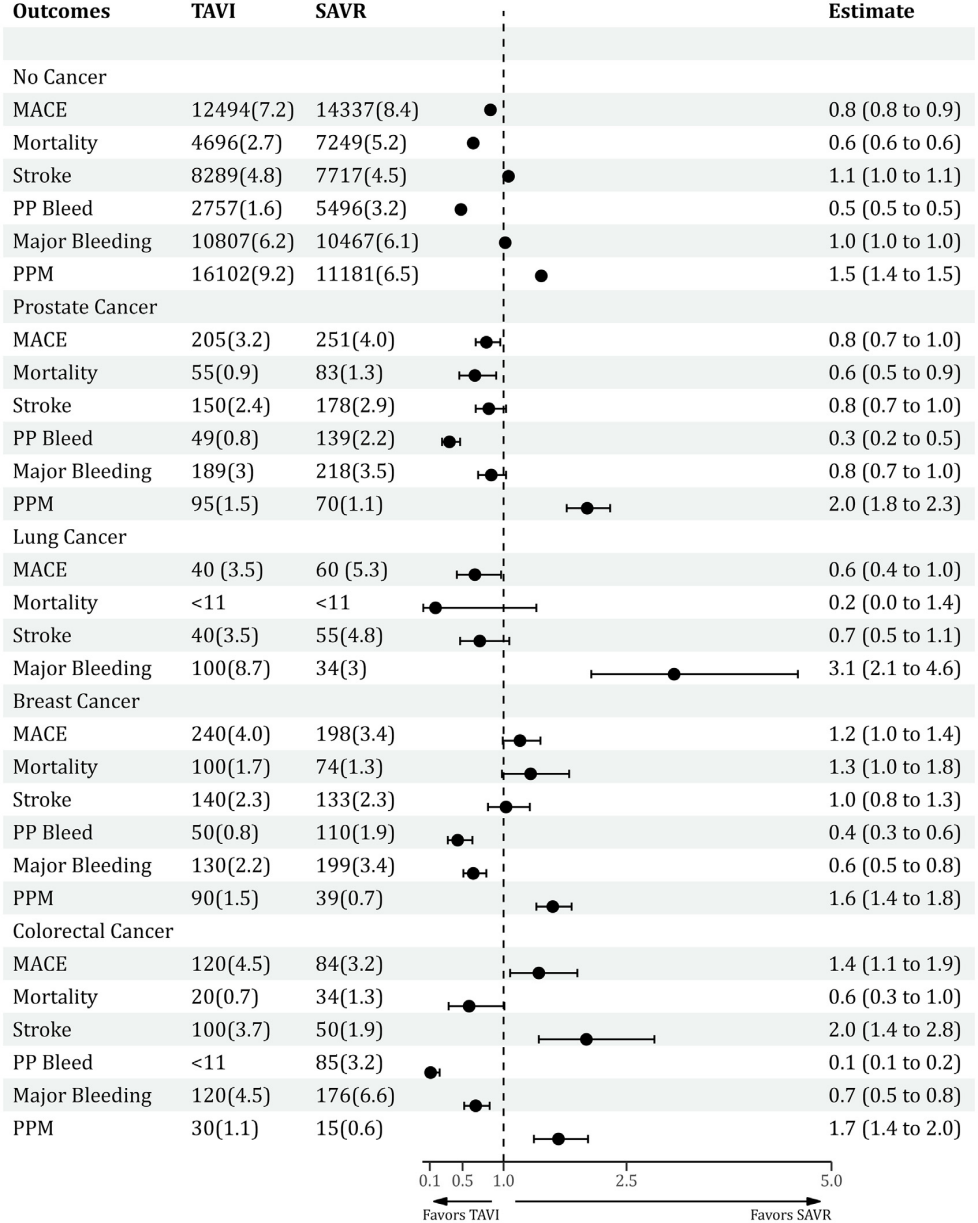
**Forest Plot Showing Adjusted OR and its 95% CI for Major Outcomes Across All Outcomes** MACE = major adverse cardiovascular events; PPM = permanent pacemaker; SAVR = surgical aortic valve replacement; TAVI = transcatheter aortic valve implantation.

Compared with SAVR, TAVI was associated with significantly lower odds of MACE in patients with lung (aOR: 0.65; 95% CI: 0.43-0.97) and prostate cancer (aOR: 0.79; 95% CI: 0.66-0.96), while a higher rate of MACE in CRC (aOR: 1.43; 95% CI: 1.08-1.90). The risk of MACE was similar in TAVI vs SAVR among breast (aOR: 1.20; 95% CI: 0.99-1.45) and renal cancer (aOR: 1.36; 95% CI: 0.84-2.2) patients (Figures 4 and 5, Central Illustration). The intergroup (TAVI vs SAVR) odds of stroke and in-hospital mortality were similar across all cancer types, except that CRC showed a higher rate of stroke (aOR: 2.01; 95% CI: 1.43-2.84), while prostate cancer had a lower risk of in-hospital mortality (aOR: 0.65; 95% CI: 0.46-0.91) with TAVI. The bivariate analysis of major components of MACE is given in Supplemental Figure 6. Among all cancer types, TAVI was associated with a lower risk of major bleeding and a higher rate of PPM implantation except in lung cancer, which showed a higher risk of major bleed (aOR: 3.08; 95% CI: 2.07-4.59) and a lower need for PPM (aOR: 0.62; 95% CI: 0.45-0.84) compared with SAVR. The risk of procedural complications was mostly similar or lower with TAVI in most of the cancer types (Table 4). Patients with CRC had a higher mean length of stay compared with other cancer types (Supplemental Figure 7).

**Central Illustration:**
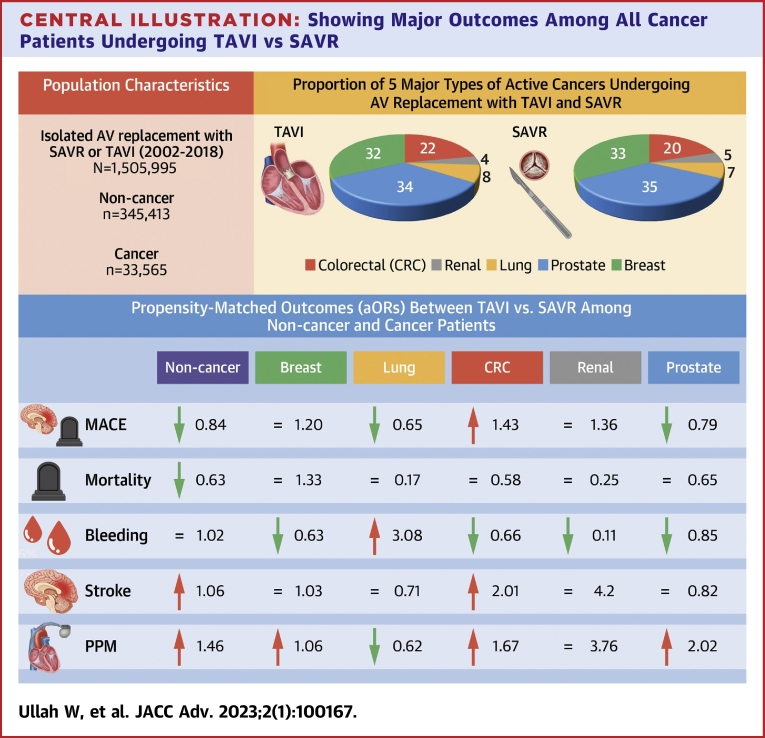
**Showing Major Outcomes Among All Cancer Patients Undergoing TAVI vs SAVR** = means no significant difference in the estimates. CRC = colorectal cancer; MACE = major adverse cardiovascular events; PPM = permanent pacemaker; SAVR = surgical aortic valve replacement; TAVI = transcatheter aortic valve implantation.

#### Sensitivity analysis of cancer patients

On sensitivity analysis, exclusion of patients with metastatic cancer, and selection of patients with active cancer-only mirrored the findings of the pooled analysis, favoring TAVI over SAVR in all cancer types (except CRC) due to a lower or similar risk of MACE and mortality. The detailed odds ratios are presented in Supplemental Table 16. We also used the aforementioned prespecified demographic groups to estimate in-hospital outcomes for each cancer type. Contrary to the net CRC odds, restricting the analysis to men only showed no significant difference in the rate of MACE and stroke with TAVI vs SAVR. Restricting the analysis to older adults with breast cancer, showed a higher rate of MACE and mortality with TAVI compared with SAVR (Supplemental Tables 17 and 18).

#### Interaction analysis of cancer patients

The interaction analysis validated the findings of sensitivity analysis by taking age and gender as potential moderators of major outcomes in TAVI vs SAVR groups (Supplemental Figures 8 to 15, Supplemental Tables 19 to 26). Females and older adults with CRC undergoing TAVI had a significantly higher risk of MACE and stroke compared with males and younger patients, respectively. TAVI in older adults with breast cancer had a significantly higher risk of MACE and mortality compared with both TAVI in younger patients as well as SAVR in older adults.

## Discussion

To our knowledge, this is the largest nationwide study comparing the outcomes of TAVI vs SAVR in patients with severe AS in conjunction with one of the most common malignancies (breast, lung, colorectal, renal, or prostate cancer). The major findings of our study are: Compared with SAVR, TAVI was associated with a similar or lower rate of MACE, stroke, and in-hospital mortality in all different types of cancers (breast, lung, prostate, and renal) except CRC. Among all cancer types, TAVI in patients with prostate cancer had the most favorable outcomes in terms of lowest mortality and MACE compared with SAVR. TAVI in patients with lung cancer had a higher risk of nonfatal major bleeding but it did not translate into higher MACE or mortality. The 43% higher odds of MACE and 2-fold increased risk of stroke with TAVI among CRC patients were entirely driven by the older and female population. Overall, the annual trend showed increased utilization of TAVI in all cancer types and a gradual decline in the complication rate except for the persistently elevated risk of the need for PPM with TAVI.

Despite recent advancements in technology, expanding the indications of TAVI to low- and intermediate-risk surgical patients, futility concerns in patients with cancer persists. Despite this, there has been a rising trend in the use of TAVI procedures in cancer patients during the last decade. The current study shows that TAVI utilization in cancer patients has now surpassed SAVR procedures. This was more pronounced among lung cancer patients with an early rise in TAVI in 2012, while a TAVI rise in all other major cancer types was observed from 2015 onward. This shift in the utilization of TAVI compared with SAVR implies increased feasibility, advancements in transcatheter technology, greater expertise, and widespread availability of the TAVI. It also implies that the off-label use of TAVI was preferred in low-risk patients with cancer.^11^ The findings of our study echo the observation by the STS-ACC TVT (Society of Thoracic Surgeons-American College of Cardiology Transcatheter Valve Therapy) registry that also indicated a gradual decline in the use of SAVR, while the portion of cancer patients among TAVI recipients increased over the years.^12^

AS management in cancer patients is very complex due to nonspecific and overlapping symptoms, higher periprocedural risks of complications, and concerns for futility in advanced stages of cancer with lower life expectancy. Previous smaller studies suggested that TAVI in cancer might be safe, with a similar risk of short-term mortality and procedural complications compared with those without cancer, however, the relative merits of TAVI vs SAVR in the full spectrum of different cancer types and different baseline characteristics were not evaluated.^13^^,^^14^ We believe that the safety of TAVI in cancer patients is contingent upon the cancer type, demographics, and underlying comorbidities. Our findings suggest that compared with SAVR, TAVI significantly reduced the overall risk of composite ischemic events in patients with cancer except those with CRC.

In the cancer patient, the highest benefits with TAVI were attained with prostate cancer as evidenced by significantly lower odds of in-hospital mortality, major bleeding, and MACE compared with SAVR. By the magnitude of the effect size, the estimated outcomes of TAVI in prostate cancer were similar to noncancer patients and were invariant across different age groups. One can argue that the use of prostate-specific antigen tests for the diagnosis of apparently localized early-stage disease might have introduced a lead-time bias, potentially classifying many healthy individuals with low-grade prostate cancer leading to these findings. An alternative explanation would be that low acuity, high chemosensitivity, and excellent response of prostate cancer to radiotherapy might have enabled favorable outcomes for these patients treated with TAVI.^15^

CRC, on the other hand, had a higher in-hospital MACE with TAVI compared with SAVR, but these findings were driven by higher odds of stroke, as there was no difference in mortality between the 2 procedures. Also, the higher MACE risk was only observed in patients >65 years, indicating that older adults, being frail, have less cardiac reserve and thus might benefit less from TAVI. Additionally, high stages of malignancy coupled with a lower life expectancy of CRC patients might be contributing to these outcomes. This contrasts with the limited data available from the case reports, which showed favorable outcomes with TAVI in patients with CRC.^16^^,^^17^ Similar trends were observed in patients with breast cancer, where older adults had a significantly higher risk of MACE and mortality compared with both younger patients undergoing TAVI, as well as those of the same age group undergoing SAVR. This could hypothetically be explained by the relatively higher incidence of prior exposure to therapeutic radiation in older adults, causing significant fibrosis and dystrophic calcifications of the aortic valve.^18^^,^^19^ Nonetheless, overall TAVI in patients with breast cancer patients had a similar risk of MACE and mortality as SAVR.

In the current study, irrespective of the type, active status, and metastasis of cancer, most of the procedure-related complications were lower with TAVI (except the need for PPM) due to the less-invasive nature of the procedure, shorter hospital stay, and lower need for general anesthesia. We observed a numerically lower rate of postprocedural and major bleeding with TAVI across all cancers except lung cancer, highlighting the safety and feasibility of the TAVI procedure in the highly coagulopathic environment of cancer. The higher bleeding risk in lung cancer could be due to the antiangiogenic properties of the chemotherapeutic agents and the innate nature of lung cancers to form cavitary lesions.^20^ Nonetheless, this did not translate into hard clinical outcomes as indicated by a lower or similar risk of MACE and in-hospital mortality between TAVI and SAVR in lung cancer patients.

These findings contrast the results of the study by Landes et al^13^ that showed higher 1-year mortality and increased bleeding (any bleeding 14.4% vs 6.9%; major bleeding 8.6% vs 3.1%) with TAVI among cancer vs noncancer patients, respectively. However, the higher mortality was mostly attributed to advanced cancer-related deaths from noncardiovascular causes, rather than TAVI-related complications. A recent meta-analysis by Murphy et al^21^ showed no difference in both short- and long-term complications and mortality between cancer and noncancer patients with TAVI reinforcing its role in cancer patients.

Overall, our analysis revealed an increased need for PPM implantation with TAVI, which was consistently high over the years from 2002 to 2018. This implies that factors other than operator skills and expertise such as inherent procedural and prosthesis-related factors might be playing a predominant role here.^22^

### Study Limitations

The findings of our study should be interpreted with the acknowledgment of certain limitations. Being a cross-sectional study, we could only report a temporal association; no definitive conclusions regarding the causality and long-term outcomes could be made. Also, it is important to note that despite using codes for possible postprocedure events, and events that can be linked with TAVI and SAVR (bleeding, PPM), we could not determine the actual timing (preprocedure or postprocedure) of these events. Data are subject to inherent selection bias as only eligible patients with cancer and severe AS were chosen for TAVI. Similarly, the influence of differences in disease assessment, documentation, and center-specific expertise on the measured outcomes could not be assessed. Lack of information regarding the echocardiographic measures, individual valvular anatomy, STS score, stages of cancer, and hemodynamics precluded further individualized risk assessment. Although all codes were validated from reliable sources, the possibility of an inadvertent error in coding cannot be entirely excluded. Due to the nature of the data, we were unable to retrieve information regarding the severity of AS, duration of intervention, type of valve used, stage of cancer, and medications used by the patients. Similarly, due to the inclusion of repeated observations from patients who underwent >1 AVR (TAVI or SAVR), a distinction between index and repeat events was not possible. Although some of the unquantifiable potential confounders such as operators’ skills and device type could not be assessed, the yearly trend analysis was performed as a surrogate marker for these covariates. A larger-scale study of a randomized population followed up on the prospective scale is needed to validate our findings.

## Conclusions

Overall, TAVI appears to be a safer alternative to SAVR in patients with breast, prostate, renal, and lung cancers due to a similar or lower risk of MACE and mortality. Females and patients >65 years old might have higher MACE with TAVI in colorectal cancer; however, there was no difference in mortality irrespective of age and sex. Due to variable outcomes, informed multifaceted decisions by the heart team in conjunction with the oncologists should be used to choose appropriate candidates for TAVI.

## Funding support and author disclosures

The authors have reported that they have no relationships relevant to the contents of this paper to disclose.

PERSPECTIVES **COMPETENCY IN MEDICAL KNOWLEDGE:** TAVI in patients with prostate, breast, lung, and renal cancer might serve as a safer alternative to SAVR due to a significantly lower or similar risk of mortality and MACE.

**TRANSLATIONAL OUTLOOK:** Further studies are needed to decide on the expansion of indications of TAVI to populations with different stages and types of cancers.
