# Supplementary material for: Trends and Outcomes of TAVI and SAVR in Cancer and Noncancer Patients: A Nationwide Analysis
Source: JACC Adv. 2023 Jan 27;2(1):100167. doi: 10.1016/j.jacadv.2022.100167 (PMC11198436; doi:10.1016/j.jacadv.2022.100167)
Supplement: Supplemental Tables S1–S26 and Figures S1–S15 [file mmc1.docx]

**Supplemental Table 1: ICD 10 codes for the major variables used in the comparison of percutaneous and surgical approaches.**

| Variable | ICD-10 codes |
| --- | --- |
| SAVR | 02RF07, 02RF07Z, 02RF08, 02RF08Z, 02RF0JZ, 02RF0KZ |
| TAVI | 02RF37, 02RF37H, 02RF37Z, 02RF38, 02RF38H, 02RF38Z, 02RF3J, 02RF3JH, 02RF3JZ, 02RF3K, 02RF3KH, 02RF3KZ, 02RF4, 02RF47, 02RF47Z, 02RF48, 02RF48Z, 02RF4J, 02RF4JZ, 02RF4K, 02RF4KZ |
| Colorectal Cancer | Prior history of cancer: Z85038, Z85048. Active Cancer: C17, C18, C20, C21. Metastatic: C785 |
| Renal Cancer | Prior history of cancer: Z85528, Z8553. Active Cancer: C64, C65, C66. Metastatic: C7900, C7901, C7902 |
| Prostate Cancer | Prior history of cancer: Z8546. Active Cancer: C61 |
| Breast Cancer | Prior history of cancer: Z853. Active Cancer: C50; Metastatic: C7981, C7981 |
| Lung Cancer | Prior history of cancer: Z85118, Active Cancer: C33, C34, C38, C39; Metastatic: C7800, C7801, C7802, C781, C782, C783, C7830, C7839 |
| Stroke | I63-I69 |
| PPM | 0JH634Z-636Z |
| PP Bleed | L7622 |

SAVR: Surgical aortic valve replacement, TAVI: transcatheter valve implantation, CS: Cardiogenic Shock, PPM: Permanent Pacemaker, PP Bleed: Postprocedure Bleed

For all these codes we identified the counterpart ICD-9 codes

**Supplemental Table 2: Definitions of the outcomes across all included patients**

| **Variable** | **Definition** |
| --- | --- |
| **Death** | All cause death including cardiovascular causes such as sudden cardiac death, death due to acute MI, heart failure or cardiogenic shock and non-cardiovascular causes. |
| **MACE** | A composite of nonfatal stroke and all cause in-hospital mortality. |
| **Stroke** | Ischemic or embolic cerebrovascular evidence on brain imaging |
| **Major Bleeding** | Intracavitary bleeding, need for transfusion |
| **Cardiogenic Shock** | Shock resulting from primary failure of the heart in its pumping function, as in myocardial infarction, severe cardiomyopathy, or mechanical obstruction or compression of the heart. |
| **Permanent Pacemaker (PPM) Implantation** | Cardiac resynchronization therapy to treat the delay in heart ventricle contractions in advanced heart failure |

MACE: major adverse cardiovascular events, PPM: permanent pacemaker

**Supplemental Table 3: Sample of matched and unmatched populations with quantitative measures of balance across difference types of cancers**

| **No cancer** | | **CRC** | | **KIDNEY** | | **Lung** | | **Prostate** | | **Breast** | |
| --- | --- | --- | --- | --- | --- | --- | --- | --- | --- | --- | --- |
| **Control** | Rx | Control | Rx | Control | Rx | Control | Rx | Control | Rx | Control | Rx |
| All |  |  |  |  |  |  |  |  |  |  |  |
| 208016 | 72198 | 1750 | 1482 | 568 | 500 | 594 | 874 | 4632 | 3564 | 3930 | 3164 |
| Matched | | |  |  |  |  |  |  |  |  |  |
| 34862 | 34862 | 538 | 538 | 148 | 148 | 230 | 230 | 1266 | 1266 | 1204 | 1204 |
| Unmatched | | |  |  |  |  |  |  |  |  |  |
| 173154 | 37336 | 1212 | 944 | 420 | 352 | 364 | 644 | 3366 | 2298 | 2726 | 1960 |
| **Balance test (Hansen and Bowers)** | | | | | | | | | | | |
| Chi square | p=value | Chi square | p=value | Chi square | p=value | Chi square | p=value | Chi square | p=value | Chi square | p=value |
| 50.625 | 0.043 | 35.983 | 33 | 0.331 | 0.957 | 38.025 | 0.214 | 59.255 | 0.003 | 50.625 | 0.043 |
| **Relative multivariate imbalance L1 (Iacus, King, & Porro)** | | | | | | | | | | | |
| Before matching | After matching | Before matching | After matching | Before matching | After matching | Before matching | After matching | Before matching | After matching | Before matching | After matching |
| 0.95 | 0.893 | 0.988 | 0.987 | 0.989 | 0.973 | 0.998 | 0.991 | 0.971 | 0.947 | 0.968 | 0.951 |

Rx: Treatment group, CRC: colorectal cancer

**Supplemental Table 4: Frequency of metastatic cancer and patients with active cancer after exclusion of patients without metastatic cancer and non-active cancer, respectively**

|  | **SAVR** |  |  | **TAVR** |  |  |
| --- | --- | --- | --- | --- | --- | --- |
|  | **Count** | **Row N %** | **Column N %** | **Count** | **Row N %** | **Column N %** |
| Metastatic Cancer | 1883 | 42.70% | 0.20% | 2529 | 57.30% | 0.60% |
| Active CRC | 1065 | 56.80% | 0.10% | 810 | 43.20% | 0.20% |
| Active Lung Cancer | 945 | 39.60% | 0.10% | 1440 | 60.40% | 0.40% |
| Active Prostate Cancer | 3810 | 56.90% | 0.40% | 2889 | 43.10% | 0.70% |
| Active Breast Cancer | 1107 | 47.60% | 0.10% | 1220 | 52.40% | 0.30% |
| Active Renal Cancer | 538 | 41.20% | 0.10% | 769 | 58.80% | 0.20% |

**Supplemental Table 5: Baseline Crude analysis and demographics of patients with cancer and without cancer (all numbers indicate percentages)**

|  | **No cancer** | | **Breast Cancer** | | **Colon Cancer** | | **Lung Cancer** | | **Renal Cancer** | |
| --- | --- | --- | --- | --- | --- | --- | --- | --- | --- | --- |
| **Variables,  No.** | **TAVI** | **SAVR** | **TAVI** | **SAVR** | **TAVI** | **SAVR** | **TAVI** | **SAVR** | **TAVI** | **SAVR** |
| Mean age,  years | 79.9 | 64.7 | 80.8 | 72.8 | 82.4 | 74.5 | 78.9 | 72.4 | 79.2 | 70.4 |
| Gender | | | | | | | | | | |
| Male | 53 | 61.8 | 1.2 | 0.9 | 56.7 | 62.2 | 54 | 58.8 | 79 | 71.6 |
| Female | 47 | 38.2 | 98.8 | 99.1 | 43.3 | 37.8 | 46 | 41.2 | 21 | 28.4 |
| Race | | | | | | | | | | |
| White | 82.7 | 70.8 | 86.3 | 78.3 | 85.7 | 75.6 | 87.6 | 80.3 | 53 | 82.8 |
| Black | 3.9 | 4.6 | 3.8 | 2.6 | 3.5 | 3.5 | 3 | 3 | 21.3 | 4.8 |
| Hispanic | 4.6 | 5.9 | 3.4 | 3.2 | 4.9 | 3.3 | 3 | 2 | 10.4 | 2.5 |
| Asian | 1.3 | 1.4 | 0.8 | 1.1 | 1.1 | 1.2 | 0.7 | 0.7 | 0 | 0.4 |
| Native American | 0.2 | 0.4 | 0.1 | 0.6 | 0.1 | 0.6 | 0 | 0.4 | - | - |
| Others | 2.5 | 2.5 | 2.4 | 2.4 | 2 | 2.2 | 1.1 | 3.1 | 0 | 1.4 |
| Elective vs non-elective admission | | | | | | | | | | |
| Non-elective | 19.7 | 24.9 | 15.5 | 15.9 | 15.7 | 18.1 | 16.7 | 19.7 | 11.6 | 19.8 |
| Elective | 80.3 | 75.1 | 84.5 | 84.1 | 84.3 | 81.9 | 83.3 | 80.3 | 88.4 | 80.2 |
| Admission | | | | | | | | | | |
| Weekday | 95.5 | 94.7 | 97.2 | 97 | 96.5 | 94.6 | 96.8 | 95.4 | 89.6 | 95.7 |
| Weekend | 4.5 | 5.3 | 2.8 | 3 | 3.5 | 5.4 | 3.2 | 4.6 | 10.4 | 4.3 |
| Comorbidities | | | | | | | | | | |
| PUD | 0.2 | 0 | 0.1 | 0 | 0.3 | 0.1 | 0.2 | 0 | - | - |
| Pulm Disease | 22.3 | 9.9 | 20.4 | 9.5 | 18.5 | 10.5 | 54.4 | 39.5 | 10.9 | 20.1 |
| Pulm Circ Dis | 5.6 | 7.4 | 3.7 | 7.3 | 2.6 | 8.6 | 3.7 | 7.5 | 10 | 4.5 |
| Anemia | 4.4 | 1.7 | 3.5 | 2.2 | 4 | 2.6 | 4.8 | 2.1 | 10.6 | 2.3 |
| Hypothyroid | 18.4 | 9.5 | 27.5 | 21 | 18.6 | 11.9 | 17.8 | 15.8 | 10.4 | 13.3 |
| Obesity | 15.3 | 8.8 | 15.4 | 8.8 | 11.9 | 7.2 | 11.2 | 7.5 | 0 | 11.9 |
| Coagulopathy | 1.5 | 4.7 | 1.5 | 2.7 | 1.2 | 3.3 | 1.1 | 3 | 0 | 2.2 |
| PeripheralAD | 7.3 | 4.7 | 4.7 | 4.2 | 5.4 | 6.2 | 6.6 | 7.7 | 0 | 5.1 |
| FHCAD | 5.9 | 4.6 | 7.9 | 5.5 | 7.3 | 5.1 | 5.3 | 4.6 | 0 | 6.6 |
| OSA | 11.8 | 7.1 | 8.6 | 5.1 | 9.3 | 5.6 | 9.4 | 10.2 | 5.3 | 15.8 |
| LD | 1.9 | 2.3 | 1.4 | 1.3 | 1.3 | 1.3 | 1.1 | 1.4 | 5.3 | 0.6 |
| Drug use | 0.8 | 8.2 | 0.6 | 3.9 | 0.4 | 3.4 | 0.2 | 7.3 | 0 | 2 |
| Smoking | 31.7 | 25.3 | 27.6 | 22 | 37.3 | 31.5 | 59.7 | 48.6 | 46.8 | 36.1 |
| Prior PCI | 5.2 | 3.3 | 4.1 | 3.6 | 7.7 | 5.1 | 5.7 | 7.1 | 10.6 | 3.7 |
| Prior CABG | 17.3 | 4.4 | 8.2 | 2.3 | 15.7 | 6.4 | 13 | 7.5 | 5.3 | 9.8 |
| HF | 72.9 | 30.4 | 70.8 | 26.7 | 69.1 | 28.1 | 71.6 | 30.8 | 42.6 | 52 |
| Prior MI | 11.9 | 3.7 | 9 | 3.5 | 12.3 | 4.5 | 11.4 | 6.1 | 10.6 | 8.5 |
| AFIB | 39.6 | 38 | 34.4 | 42.8 | 40.1 | 45.1 | 31.4 | 43.3 | 42.2 | 41.4 |
| AFlutter | 4 | 5.8 | 2.6 | 4.4 | 3.1 | 5.7 | 3.7 | 6.5 | 5.3 | 4.3 |
| MS | 0.2 | 0.1 | 0.3 | 0.1 | 0.3 | 0 | 0.2 | 0 | 5.3 | 0 |
| CAD | 67.8 | 29.9 | 57.1 | 28.9 | 70.7 | 37.6 | 64.7 | 45.2 | 62.7 | 50.5 |
| ESRD | 4.4 | 3.2 | 1.1 | 0.8 | 3 | 1.5 | 1.6 | 1.2 | 10.6 | 10.5 |
| Dyslipidemia | 66.6 | 44.4 | 66.9 | 55.8 | 67.6 | 53.4 | 69.6 | 50.8 | 62.7 | 59.6 |
| HTN | 12.1 | 42 | 12 | 51.9 | 10.1 | 44.2 | 9.1 | 46.1 | 20.6 | 17.6 |
| DM | 27.6 | 5.3 | 23.2 | 6.5 | 28.5 | 6.7 | 23.6 | 5.9 | 26.6 | 19.6 |

PUD: peptic ulcer disease, PulmDisease: pulmonary disease, PulmCircDis: pulmonary circulatory disease, PeripheralAD: peripheral artery disease, FHCAD: family history of coronary artery disease, OSA, obstructive sleep apnea, LD: lung disease, PCI: percutaneous coronary intervention, CABG: coronary artery bypass graft, HF: heart failure, MI: myocardial infarction, AFIB: atrial fibrillation, MS: mitral stenosis, CAD: coronary artery disease, ESRD: end stage renal disease, HTN: hypertension, DM: diabetes mellitus.

**Supplemental Table 6: Yearly trend of valve replacement among different cancer groups**

|  | **All Cancer** | **CRC** | **Renal** | **Lung** | **Prostate** | **Breast** |
| --- | --- | --- | --- | --- | --- | --- |
| **2002** | 2527 (6.10%) | 341 (0.80%) | 47 (0.10%) | 130 (0.30%) | 753 (1.80%) | 709 (1.70%) |
| **2003** | 2670 (5.80%) | 334 (0.70%) | 37 (0.10%) | 130 (0.30%) | 568 (1.20%) | 740 (1.60%) |
| **2004** | 2681 (6.00%) | 276 (0.60%) | 78 (0.20%) | 108 (0.20%) | 701 (1.60%) | 761 (1.70%) |
| **2005** | 3067 (6.10%) | 261 (0.50%) | 59 (0.10%) | 125 (0.30%) | 977 (2.00%) | 879 (1.80%) |
| **2006** | 3382 (6.20%) | 410 (0.70%) | 98 (0.20%) | 136 (0.20%) | 984 (1.80%) | 880 (1.60%) |
| **2007** | 3705 (6.70%) | 547 (1.00%) | 118 (0.20%) | 157 (0.30%) | 840 (1.50%) | 1090 (2.00%) |
| **2008** | 4014 (6.80%) | 622 (1.10%) | 116 (0.20%) | 164 (0.30%) | 1276 (2.20%) | 766 (1.30%) |
| **2009** | 5341 (7.20%) | 646 (0.90%) | 203 (0.30%) | 165 (0.20%) | 1733 (2.30%) | 1293 (1.70%) |
| **2010** | 4048 (6.20%) | 430 (0.70%) | 210 (0.30%) | 169 (0.30%) | 1134 (1.70%) | 1042 (1.60%) |
| **2011** | 6362 (8.00%) | 785 (1.00%) | 312 (0.40%) | 375 (0.50%) | 1769 (2.20%) | 1631 (2.10%) |
| **2012** | 6910 (7.60%) | 740 (0.80%) | 260 (0.30%) | 330 (0.40%) | 1970 (2.20%) | 1850 (2.00%) |
| **2013** | 8410 (7.80%) | 1000 (0.90%) | 240 (0.20%) | 330 (0.30%) | 2390 (2.20%) | 2090 (1.90%) |
| **2014** | 9720 (8.00%) | 960 (0.80%) | 330 (0.30%) | 410 (0.30%) | 2810 (2.30%) | 2530 (2.10%) |
| **2015** | 10910 (8.30%) | 1330 (1.00%) | 440 (0.30%) | 470 (0.40%) | 3310 (2.50%) | 2890 (2.20%) |
| **2016** | 17590 (11.90%) | 2150 (1.50%) | 900 (0.60%) | 1340 (0.90%) | 5670 (3.80%) | 4710 (3.20%) |
| **2017** | 19790 (12.20%) | 2500 (1.50%) | 890 (0.50%) | 1310 (0.80%) | 6590 (4.00%) | 5370 (3.30%) |
| **2018** | 21050 (12.20%) | 2620 (1.50%) | 950 (0.60%) | 1420 (0.80%) | 7010 (4.10%) | 5780 (3.40%) |

CRC: colorectal cancer

**Supplemental Table 7: Yearly trend of SAVR and TAVI among no cancer and different cancers (all numbers are in percentages)**

|  | **No Cancer** | | **Prostate** | | | **Lung** | | | **Kidney** | | | **CRC** | | | **Breast** | | |  |
| --- | --- | --- | --- | --- | --- | --- | --- | --- | --- | --- | --- | --- | --- | --- | --- | --- | --- | --- |
| **Year** | SAVR | TAVI | | SAVR | TAVI | | SAVR | TAVI | | SAVR | TAVI | | SAVR | TAVI | | SAVR | TAVI | |
| **2011** | 98 | 2 | | 96 | 4 | | 98 | 2 | | 100 | 0 | | 96 | 4 | | 98 | 2 | |
| **2012** | 86 | 14 | | 82 | 18 | | 58 | 42 | | 80 | 20 | | 79 | 21 | | 81 | 19 | |
| **2013** | 78 | 22 | | 74 | 26 | | 46 | 54 | | 75 | 25 | | 70 | 30 | | 67 | 33 | |
| **2014** | 70 | 30 | | 66 | 34 | | 41 | 59 | | 60 | 40 | | 53 | 47 | | 57 | 43 | |
| **2015** | 62 | 38 | | 52 | 48 | | 29 | 71 | | 54 | 46 | | 39 | 61 | | 49 | 51 | |
| **2016** | 49 | 51 | | 32 | 68 | | 12 | 88 | | 26 | 74 | | 27 | 73 | | 30 | 70 | |
| **2017** | 40 | 60 | | 25 | 75 | | 20 | 80 | | 29 | 71 | | 21 | 79 | | 23 | 77 | |
| **2018** | 36 | 64 | | 24 | 76 | | 12 | 87 | | 18 | 82 | | 18 | 82 | | 17 | 83 | |

CRC: colorectal cancer, TAVI: transcatheter valve implantation, SAVR: surgical aortic valve replacement

**Supplemental Table 8: Proportion of different outcomes between TAVI and SAVR over the years among no cancer and other cancers**

| **Year** | **Cancer** | **Compare** | **MACE** | **Mortality** | **PPM** | **Stroke** | **Major Bleeding** |
| --- | --- | --- | --- | --- | --- | --- | --- |
| **2011** | No Cancer | SAVR | 10.9% | 4.3% | 6.5% | 6.9% | 10.3% |
|  |  | TAVI | 13.8% | 2.7% | 14.4% | 10.3% | 18.4% |
|  | Breast Cancer | SAVR | 3.9% | 2.0% | 10.9% | 2.0% | 10.8% |
|  |  | TAVI | 0.0% | 0.0% | 63.6% | 0.0% | 0.0% |
|  | Colorectal Cancer | SAVR | 4.8% | 0.0% | 10.1% | 4.8% | 13.7% |
|  |  | TAVI | 0.0% | 0.0% | 0.0% | 0.0% | 0.0% |
|  | Renal Cancer | SAVR | 0.0% | 0.0% | 0.0% | 0.0% | 42.5% |
|  |  | TAVI | 0.0% | 0.0% | 0.0% | 0.0% | 0.0% |
|  | Lung Cancer | SAVR | 0.0% | 0.0% | 14.6% | 0.0% | 7.1% |
|  |  | TAVI | 0.0% | 0.0% | 0.0% | 0.0% | 0.0% |
|  | Prostate Cancer | SAVR | 4.6% | 0.0% | 7.2% | 4.6% | 1.2% |
|  |  | TAVI | 0.0% | 0.0% | 0.0% | 0.0% | 19.8% |
| **2012** | No Cancer | SAVR | 11.9% | 5.1% | 6.4% | 7.1% | 9.4% |
|  |  | TAVI | 13.6% | 5.7% | 8.8% | 7.8% | 14.8% |
|  | Breast Cancer | SAVR | 8.5% | 0.0% | 2.8% | 5.6% | 0.0% |
|  |  | TAVI | 6.9% | 0.0% | 6.9% | 3.4% | 3.4% |
|  | Colorectal Cancer | SAVR | 6.9% | 6.9% | 6.9% | 0.0% | 17.2% |
|  |  | TAVI | 10.0% | 10.0% | 0.0% | 0.0% | 20.0% |
|  | Renal Cancer | SAVR | 0.0% | 0.0% | 0.0% | 0.0% | 0.0% |
|  |  | TAVI | 40.0% | 0.0% | 0.0% | 40.0% | 0.0% |
|  | Lung Cancer | SAVR | 14.3% | 0.0% | 0.0% | 14.3% | 14.3% |
|  |  | TAVI | 11.1% | 0.0% | 11.1% | 11.1% | 0.0% |
|  | Prostate Cancer | SAVR | 7.4% | 2.9% | 5.9% | 4.4% | 13.2% |
|  |  | TAVI | 14.8% | 0.0% | 3.7% | 14.8% | 7.4% |
| **2013** | No Cancer | SAVR | 13.2% | 3.8% | 7.5% | 9.2% | 8.6% |
|  |  | TAVI | 13.2% | 4.4% | 9.8% | 8.2% | 12.0% |
|  | Breast Cancer | SAVR | 10.6% | 0.0% | 10.6% | 10.6% | 4.3% |
|  |  | TAVI | 12.2% | 4.1% | 12.2% | 8.2% | 4.1% |
|  | Colorectal Cancer | SAVR | 0.0% | 0.0% | 5.3% | 0.0% | 21.1% |
|  |  | TAVI | 12.5% | 0.0% | 16.7% | 12.5% | 8.3% |
|  | Renal Cancer | SAVR | 0.0% | 0.0% | 0.0% | 0.0% | 0.0% |
|  |  | TAVI | 0.0% | 0.0% | 25.0% | 0.0% | 0.0% |
|  | Lung Cancer | SAVR | 22.2% | 0.0% | 22.2% | 22.2% | 0.0% |
|  |  | TAVI | 0.0% | 0.0% | 0.0% | 0.0% | 20.0% |
|  | Prostate Cancer | SAVR | 10.9% | 0.0% | 1.8% | 7.3% | 7.3% |
|  |  | TAVI | 2.2% | 2.2% | 13.3% | 0.0% | 8.9% |
| **2014** | No Cancer | SAVR | 10.8% | 4.3% | 6.1% | 6.4% | 9.5% |
|  |  | TAVI | 13.2% | 3.8% | 11.6% | 9.1% | 10.7% |
|  | Breast Cancer | SAVR | 3.3% | 0.0% | 8.2% | 3.3% | 0.0% |
|  |  | TAVI | 5.7% | 1.1% | 13.8% | 4.6% | 2.3% |
|  | Colorectal Cancer | SAVR | 0.0% | 0.0% | 3.2% | 0.0% | 3.2% |
|  |  | TAVI | 5.7% | 0.0% | 11.4% | 5.7% | 8.6% |
|  | Renal Cancer | SAVR | 20.0% | 0.0% | 0.0% | 20.0% | 0.0% |
|  |  | TAVI | 0.0% | 0.0% | 11.1% | 0.0% | 0.0% |
|  | Lung Cancer | SAVR | 0.0% | 0.0% | 0.0% | 0.0% | 0.0% |
|  |  | TAVI | 0.0% | 0.0% | 6.3% | 0.0% | 12.5% |
|  | Prostate Cancer | SAVR | 5.8% | 0.0% | 0.0% | 2.9% | 5.8% |
|  |  | TAVI | 7.2% | 1.4% | 11.6% | 4.3% | 5.8% |
| **2015** | No Cancer | SAVR | 7.7% | 3.2% | 5.3% | 4.4% | 3.9% |
|  |  | TAVI | 10.9% | 2.2% | 10.1% | 8.1% | 8.7% |
|  | Breast Cancer | SAVR | 3.5% | 0.0% | 1.2% | 1.2% | 1.2% |
|  |  | TAVI | 9.3% | 4.7% | 7.0% | 4.7% | 3.5% |
|  | Colorectal Cancer | SAVR | 2.8% | 0.0% | 0.0% | 2.8% | 5.6% |
|  |  | TAVI | 11.6% | 0.0% | 18.6% | 9.3% | 4.7% |
|  | Renal Cancer | SAVR | 0.0% | 0.0% | 0.0% | 0.0% | 7.1% |
|  |  | TAVI | 28.6% | 0.0% | 14.3% | 28.6% | 0.0% |
|  | Lung Cancer | SAVR | 0.0% | 0.0% | 0.0% | 0.0% | 16.7% |
|  |  | TAVI | 7.7% | 0.0% | 7.7% | 7.7% | 0.0% |
|  | Prostate Cancer | SAVR | 7.8% | 3.3% | 4.4% | 4.4% | 5.6% |
|  |  | TAVI | 6.5% | 0.0% | 12.9% | 6.5% | 4.3% |
| **2016** | No Cancer | SAVR | 4.6% | 2.2% | 5.9% | 1.2% | 1.0% |
|  |  | TAVI | 4.9% | 2.5% | 8.9% | 1.7% | 2.7% |
|  | Breast Cancer | SAVR | 2.9% | 0.0% | 5.2% | 1.7% | 0.6% |
|  |  | TAVI | 2.0% | 1.0% | 10.7% | 0.0% | 1.0% |
|  | Colorectal Cancer | SAVR | 0.0% | 0.0% | 7.6% | 0.0% | 0.0% |
|  |  | TAVI | 4.5% | 2.3% | 4.5% | 0.0% | 6.8% |
|  | Renal Cancer | SAVR | 6.1% | 6.1% | 0.0% | 0.0% | 3.0% |
|  |  | TAVI | 0.0% | 0.0% | 5.9% | 0.0% | 0.0% |
|  | Lung Cancer | SAVR | 10.0% | 0.0% | 40.0% | 0.0% | 0.0% |
|  |  | TAVI | 8.3% | 0.0% | 4.2% | 8.3% | 4.2% |
|  | Prostate Cancer | SAVR | 3.0% | 0.0% | 3.9% | 0.0% | 0.0% |
|  |  | TAVI | 1.9% | 0.0% | 10.0% | 0.0% | 0.0% |
| **2017** | No Cancer | SAVR | 3.7% | 2.2% | 5.4% | 1.1% | 1.0% |
|  |  | TAVI | 3.6% | 1.7% | 7.3% | 1.0% | 1.4% |
|  | Breast Cancer | SAVR | 2.4% | 2.4% | 4.2% | 0.0% | 0.0% |
|  |  | TAVI | 5.5% | 0.9% | 10.0% | 0.9% | 0.9% |
|  | Colorectal Cancer | SAVR | 9.7% | 4.8% | 8.1% | 1.6% | 0.0% |
|  |  | TAVI | 3.8% | 0.0% | 7.6% | 1.9% | 0.0% |
|  | Renal Cancer | SAVR | 0.0% | 0.0% | 2.6% | 0.0% | 0.0% |
|  |  | TAVI | 0.0% | 0.0% | 14.3% | 0.0% | 0.0% |
|  | Lung Cancer | SAVR | 0.0% | 0.0% | 18.2% | 0.0% | 0.0% |
|  |  | TAVI | 0.0% | 0.0% | 10.5% | 0.0% | 15.8% |
|  | Prostate Cancer | SAVR | 1.1% | 1.1% | 10.4% | 0.0% | 0.5% |
|  |  | TAVI | 3.9% | 1.8% | 10.8% | 0.7% | 0.0% |
| **2018** | No Cancer | SAVR | 5.3% | 2.5% | 6.5% | 1.0% | 0.8% |
|  |  | TAVI | 4.3% | 1.7% | 8.4% | 1.3% | 1.7% |
|  | Breast Cancer | SAVR | 1.6% | 1.6% | 5.7% | 0.0% | 0.8% |
|  |  | TAVI | 2.1% | 0.7% | 7.9% | 0.0% | 2.1% |
|  | Colorectal Cancer | SAVR | 4.3% | 0.0% | 13.0% | 4.3% | 0.0% |
|  |  | TAVI | 0.0% | 0.0% | 11.1% | 0.0% | 0.0% |
|  | Renal Cancer | SAVR | 0.0% | 0.0% | 7.1% | 0.0% | 14.3% |
|  |  | TAVI | 0.0% | 0.0% | 5.6% | 0.0% | 0.0% |
|  | Lung Cancer | SAVR | 8.0% | 0.0% | 8.0% | 8.0% | 0.0% |
|  |  | TAVI | 0.0% | 0.0% | 4.2% | 0.0% | 8.3% |
|  | Prostate Cancer | SAVR | 2.3% | 0.0% | 6.9% | 1.1% | 1.1% |
|  |  | TAVI | 3.3% | 0.7% | 11.4% | 0.7% | 2.7% |

MACE: Major Adverse Cardiovascular Events; PPM: Permanent Pacemaker;  SAVR: surgical aortic valve replacement, TAVI: transcatheter valve implantation

**Supplemental Table 9: Propensity matched estimates of different outcomes between TAVI and SAVR over the years among no cancer and other cancers**

| **Outcomes** | **2012** | **2013** | **2014** | **2015** | **2016** | **2017** | **2018** |
| --- | --- | --- | --- | --- | --- | --- | --- |
| **No Cancer** | |  |  |  |  |  |  |
| MACE | 1.17(1.08-1.27) | 1.00(0.93-1.07) | 1.26(1.17-1.36) | 1.46(1.36-1.57) | 1.06(0.98-1.15) | 0.96(0.87-1.05) | 0.81(0.75-0.88) |
| Mortality | 1.13(1.00-1.29) | 1.19(1.05-1.35) | 0.87(0.77-0.98) | 0.67(0.60-0.76) | 1.15(1.0-1.30) | 0.77(0.68-0.88) | 0.69(0.61-0.78) |
| Bleeding | 1.68(1.54-1.84) | 1.45(1.33-1.57) | 1.15(1.06-1.24) | 2.31(2.10-2.55) | 2.60(2.25-3.01) | 1.46(1.23-1.72) | 2.15(1.80-2.58) |
| PPM | 1.40(1.25-1.55) | 1.34(1.22-1.46) | 2.01(1.83-2.21) | 1.99(1.84-2.18) | 1.57(1.46-1.68) | 1.38(1.28-1.49) | 1.32(1.23-1.41) |
| Stroke | 1.11(0.99-1.23) | 0.88(0.81-0.96) | 1.46(1.33-1.60) | 1.93(1.76-2.12) | 1.40(1.21-1.63) | 0.89(0.75-1.06) | 1.36(1.14-1.62) |
| **Breast** |  |  |  |  |  |  |  |
| MACE | 0.80(0.45-1.45) | 1.17(0.72-1.92) | 1.80(0.90-3.60) | 2.80(1.60-4.93) | 0.69(0.38-1.26) | 2.33(1.40-3.90) | 1.33(0.64-2.73) |
| Mortality | - | - | - | - | - | 0.37(0.17-0.80) | 0.44(0.18-1.05) |
| Bleeding | - | 0.96(0.44-2.08) | - | 3.04(1.17-7.88) | 1.75(0.60-5.15) | - | 2.67(1.03-6.92) |
| PPM | 2.56(1.18-5.56) | 1.17(0.72-1.92) | 1.79(1.14-2.82) | 6.30(2.51-15.8) | 2.16(1.51-3.10) | 2.52(1.70-3.73) | 1.41(0.95-2.09) |
| Stroke | 0.60(0.28-1.30) | 0.75(0.44-1.26) | 1.42(0.70-2.88) | 4.10(1.61-10.4) | - | - | - |
| **CRC** |  |  |  |  |  |  |  |
| MACE | 1.50(1.1-3.75) | - | - | 4.61(1.81-11.7) | - | 0.37(0.21-0.66) | - |
| Mortality | 1.50(0.60-3.75) | - | - | - | - | - | - |
| Bleeding | 1.20(0.63-2.30) | 0.34(0.19-0.61) | 2.81(1.07-7.39) | 0.83(0.38-1.81) | - | - | - |
| PPM | - | 3.60(1.75-7.41) | 3.87(1.50-10) | - | 0.58(0.32-1.04) | 0.94(0.56-1.58) | 0.83(0.53-1.32) |
| Stroke | - | - | - | 3.59(1.39-9.25) | - | 1.18(0.40-3.50) | - |
| **Prostate** |  |  |  |  |  |  |  |
| MACE | 2.19(1.29-3.72) | 0.19(0.09-0.39) | 1.27(0.74-2.17) | 0.82(0.53-1.26) | 0.64(0.36-1.13) | 3.72(1.88-7.33) | 1.48(0.88-2.50) |
| Mortality | - | - | - | - | - | 1.65(0.79-3.46) | - |
| Bleeding | 0.52(0.30-0.91) | 1.24(0.71-2.18) | 1.00(0.58-1.74) | 0.76(0.46-1.28) | - | - | 2.38(1.18-4.78) |
| PPM | 0.62(0.28-1.34) | 8.31(3.29-20.96) | - | 3.19(1.96-5.19) | 2.74(1.88-3.98) | 1.04(0.79-1.37) | 1.74(1.28-2.37) |
| Stroke | 3.77(2.03-6.98) | - | 1.52(0.74-3.15) | 1.48(0.88-2.49) | - | - | 0.58(0.24-1.41) |

CRC: colorectal cancer, MACE: Major Adverse Cardiovascular Events; PPM: Permanent Pacemaker.

**Supplemental Table 10: Unadjusted and propensity adjusted odds of outcomes among non cancer patients between TAVI and SAVR**

| **No Cancer** | **Crude Analysis** | | | **Propensity Matched** | | |
| --- | --- | --- | --- | --- | --- | --- |
| **Variable** | TAVI | SAVR | uOR | TAVI | SAVR | aOR |
| **MACE** | 18845(5.2) | 72749(7.2) | 0.71 (0.70-0.72) | 12494(7.2) | 14337(8.4) | 0.84 (0.82-0.87) |
| **Mortality** | 7550(2.1) | 31676(3.1) | 0.66 (0.64-0.69) | 4696(2.7) | 7249(5.2) | 0.63 (0.60-0.65) |
| **Stroke** | 12047(3.3) | 44291(4.4) | 0.75 (0.74-0.77) | 8289(4.8) | 7717(4.5) | 1.06 (1.02-1.09) |
| **PP Bleed** | 4022(1.1) | 43145(4.3) | 0.25 (0.24-0.26) | 2757(1.6) | 5496(3.2) | 0.48 (0.46-0.51) |
| **Major Bleeding** | 15626(4.3) | 72285(7.1) | 0.59 (0.58-0.60) | 10807(6.2) | 10467(6.1) | 1.02 (0.99-1.04) |
| **PPM** | 33734(9.3) | 55910(5.5) | 1.76 (1.74-1.79) | 16102(9.2) | 11181(6.5) | 1.46 (1.42-1.49) |

MACE: Major Adverse Cardiovascular Events; PP: Post-procedure; CS: Cardiogenic Shock; PPM: Permanent Pacemaker; PVAD: Percutaneous Ventricular Assist Device; IABP: Intra-Aortic Balloon Pump; ECMO: ExtraCorporeal Membrane Oxygenation. HD: Hemodialysis

**Supplemental Table 11: Unadjusted and propensity adjusted odds ratio among prostate cancer patients between TAVI and SAVR**

| **Prostate** | **Crude Analysis** | | | **Propensity Matched** | | |
| --- | --- | --- | --- | --- | --- | --- |
| **Variable** | TAVI | SAVR | uOR | TAVI | SAVR | aOR |
| **MACE** | 390(2.2) | 1019(4.5) | 0.48 (0.42-0.54) | 205(3.2) | 251(4.0) | 0.79 (0.66-0.96) |
| **Mortality** | 160(0.9) | 271(1.2) | 0.75 (0.62-0.91) | 55(0.9) | 83(1.3) | 0.65 (0.46-0.91) |
| **Stroke** | 230(1.3) | 778(3.4) | 0.37 (0.32-0.43) | 150(2.4) | 178(2.9) | 0.82 (0.66-1.03) |
| **PP Bleed** | 89(0.5) | 756(3.3) | 0.15 (0.12-0.18) | 49(0.8) | 139(2.2) | 0.34 (0.25-0.47) |
| **Major Bleeding** | 379(2.1) | 1189(5.2) | 0.39 (0.35-0.44) | 189(3) | 218(3.5) | 0.85 (0.69-0.99) |
| **PPM** | 180(1) | 119(0.5) | 1.93 (1.53-2.44) | 95(1.5) | 70(1.1) | 2.02 (1.77-2.30) |

MACE: Major Adverse Cardiovascular Events; PP: Post-procedure; CS: Cardiogenic Shock; PPM: Permanent Pacemaker; PVAD: Percutaneous Ventricular Assist Device; HD: Hemodialysis

**Supplemental Table 12: Unadjusted and propensity adjusted odds ratio among lung cancer patients between TAVI and SAVR**

| **Lung** | **Crude Analysis** | | | **Propensity Matched** | | |
| --- | --- | --- | --- | --- | --- | --- |
| **Variable** | TAVI | SAVR | uOR | TAVI | SAVR | aOR |
| **MACE** | 100(2.3) | 147(5.1) | 0.44 (0.34-0.57) | 40 (3.5) | 60 (5.3) | 0.65 (0.43-0.97) |
| **Mortality** | 50(1.1) | 37(1.3) | 0.90 (0.58-1.37) | <11 | <11 | 0.17 (0.02-1.4) |
| **Stroke** | 60(1.4) | 127(4.4) | 0.30 (0.22-0.41) | 40(3.5) | 55(4.8) | 0.71 (0.47-1.07) |
| **Major Bleeding** | 140(3.2) | 68(2.3) | 1.38 (1.03-1.85) | 100(8.7) | 34(3) | 3.08 (2.07-4.59) |

MACE: Major Adverse Cardiovascular Events; PP: Post-procedure; CS: Cardiogenic Shock; PPM: Permanent Pacemaker; PVAD: Percutaneous Ventricular Assist Device; HD: Hemodialysis

**Supplemental Table 13: Unadjusted and propensity adjusted odds ratio among breast cancer patients between TAVI and SAVR**

| **Breast** | **Crude Analysis** | | | **Propensity Matched** | | |
| --- | --- | --- | --- | --- | --- | --- |
| **Variable** | TAVI | SAVR | uOR | TAVI | SAVR | aOR |
| **MACE** | 510(3.2) | 686(3.6) | 0.90 (0.80-1.01) | 240(4.0) | 198(3.4) | 1.20 (0.99-1.45) |
| **Mortality** | 280(1.8) | 210(1.1) | 1.63 (1.36-1.95) | 100(1.7) | 74(1.3) | 1.33 (0.98-1.80) |
| **Stroke** | 240(1.5) | 512(2.7) | 0.56 (0.48-0.66) | 140(2.3) | 133(2.3) | 1.03 (0.81-1.32) |
| **PP Bleed** | 80(0.5) | 558(2.9) | 0.17 (0.13-0.21) | 50(0.8) | 110(1.9) | 0.44 (0.32-0.62) |
| **Major Bleeding** | 260(1.6) | 805(4.2) | 0.38 (0.33-0.44) | 130(2.2) | 199(3.4) | 0.63(0.51-0.79) |
| **PPM** | 220(1.4) | 127(0.7) | 2.12  (1.70-2.64) | 90(1.5) | 39(0.7) | 1.60 (1.40-1.83) |

MACE: Major Adverse Cardiovascular Events; PP: Post-procedure; CS: Cardiogenic Shock; PPM: Permanent Pacemaker; PVAD: Percutaneous Ventricular Assist Device;  ECMO: ExtraCorporeal Membrane Oxygenation, HD: Hemodialysis

**Supplemental Table 14: Unadjusted and propensity adjusted odds ratio among colorectal cancer patients between TAVI and SAVR**

| **CRC** | **Crude Analysis** | |  | **Propensity Matched** | |  |
| --- | --- | --- | --- | --- | --- | --- |
| **Variable** | TAVI | SAVR | uOR | TAVI | SAVR | aOR |
| **MACE** | 210(2.8) | 294(3.4) | 0.82 (0.68-0.98) | 120(4.5) | 84(3.2) | 1.43 (1.08-1.90) |
| **Mortality** | 100(1.3) | 114(1.3) | 1.01 (0.77-1.33) | 20(0.7) | 34(1.3) | 0.58 (0.33-1.01) |
| **Stroke** | 120(1.6) | 179(2.1) | 0.77 (0.61-0.97) | 100(3.7) | 50(1.9) | 2.01 (1.43-2.84) |
| **PP Bleed** | 20(0.3) | 272(3.2) | 0.08  (0.05-0.13) | <11 | 85(3.2) | 0.11 (0.06-0.22) |
| **Major Bleeding** | 180(2.4) | 481(5.6) | 0.42 (0.35-0.50) | 120(4.5) | 176(6.6) | 0.66 (0.52-0.83) |
| **PPM** | 60(0.8) | 31(0.4) | 2.24  (1.45-3.46) | 30(1.1) | 15(0.6) | 1.67 (1.37-2.03) |

MACE: Major Adverse Cardiovascular Events; PP: Post-procedure; CS: Cardiogenic Shock; PPM: Permanent Pacemaker; PVAD: Percutaneous Ventricular Assist Device;  ECMO: ExtraCorporeal Membrane Oxygenation, HD: Hemodialysis

**Supplemental Table 15: Unadjusted and propensity adjusted odds ratio among renal cancer patients between TAVI and SAVR**

| **Renal** | **Crude Analysis** | | | **Propensity Matched** | | |
| --- | --- | --- | --- | --- | --- | --- |
| **Variable** | TAVI | SAVR | uOR | TAVI | SAVR | aOR |
| **MACE** | 70(2.8) | 108(3.9) | 0.72 (0.53-0.97) | 30(4.1) | 40(5.5) | 1.36 (0.84-2.21) |
| **Mortality** | 20(0.8) | 50(1.8) | 0.44 (0.26-0.75) | - | <11 | 0.25 (0.28-2.2) |
| **Stroke** | 60(2.4) | 58(2.1) | 1.16  (0.80-1.67) | <11 | <11 | 4.17 (0.87-19.98) |
| **PP Bleed** | 30(1.2) | 95(3.4) | 0.34 (0.23-0.52) | - | <11 | ----- |
| **Major Bleed** | 80(3.2) | 195(7.0) | 0.44 (0.34-0.57) | <11 | <11 | 0.11 (0.01-0.88) |
| **PPM** | 260(10.4) | 159(5.7) | 1.92 (1.56-2.35) | 14(9.5) | <11 | 3.76 (1.21-11.71) |

MACE: Major Adverse Cardiovascular Events; PP: Post-procedure; CS: Cardiogenic Shock; PPM: Permanent Pacemaker;  ECMO: ExtraCorporeal Membrane Oxygenation, IABP: Intra-Aortic Balloon Pump, HD: Hemodialysis

**Supplemental Table 16: Sensitivity analysis by exclusion of patients with metastatic cancer and those with prior cancer. The odds ratios of TAVI vs. SAVR of non-metastatic and active cancer only.**

|  | **Breast Cancer** |  | **Colorectal Cancer** |  |
| --- | --- | --- | --- | --- |
| **Variable** | Non-Metastatic | Active Cancer | Non-Metastatic | Active Cancer |
| **MACE** | 1.22 (0.98-1.47) | 0.92 (0.62-1.38) | 1.45 (1.09-1.92) | 1.02 (1.01-1.61) |
| **Mortality** | 1.35 (0.99-1.83) | 0.91 (0.38-2.19) | 0.58 (0.34-1.02) | 0.68 (0.43-1.01) |
| **Stroke** | 1.05 (0.83-1.34) | 0.93 (0.59-1.45) | 2.04 (1.44-2.87) | 0.63 (0.37-1.09) |
| **PPM** | 1.59 (1.38-1.82) | 2.14 (1.62-2.81) | 1.46 (1.42-1.49) | 3.04 (2.08-4.44) |
|  | **Lung Cancer** |  | **Renal Cancer** |  |
| **Variable** | Non-Metastatic | Active Cancer | Non-Metastatic | Active Cancer |
| **MACE** | 0.65 (0.43-0.97) | 0.35 (0.26-0.48) | 1.45 (0.89-2.36) | 0.39 (0.24-1.3) |
| **Mortality** | 0.18 (0.01-1.5) | 0.35 (0.23-1.05) | 0.26 (0.28-2.3) | 0.70 (0.29-1.68) |
| **Stroke** | 0.71 (0.47-1.07) | 0.40 (0.25-1.2) | 4.40 (2.18-8.87) | 0.49 (0.30-0.80) |
| **PPM** | 0.61 (0.45-0.83) | 2.11 (0.98-3.06) | 4.20 (2.50-7.05) | 1.46 (1.02-2.09) |
|  | **Prostate Cancer** |  |  |  |
| **Variable** | Non-Metastatic | Active Cancer |  |  |
| **MACE** | 0.82 (0.68-0.99) | 0.75 (0.60-0.95) |  |  |
| **Mortality** | 0.73 (0.51-0.98) | 0.89 (0.72-0.99) |  |  |
| **Stroke** | 0.87 (0.69-1.08) | 0.77 (0.69-1.01) |  |  |
| **PPM** | 2.00 (1.75-2.29) | 1.01 (1.00-1.09) |  |  |

**Supplemental Table 17: Proportion of different outcomes between TAVI and SAVR across different gender and age among no cancer and different cancers**

| **Cancer** |  | **Compare** | **MACE** | **Mortality** | **Stroke** | **Bleeding** | **PPM** |
| --- | --- | --- | --- | --- | --- | --- | --- |
| **Gender** |  |  |  |  |  |  |  |
| **No cancer** | Male | SAVR | 7.6% | 3.6% | 4.3% | 6.5% | 6.2% |
|  |  | TAVI | 6.7% | 2.5% | 4.5% | 6.5% | 9.6% |
|  | Female | SAVR | 9.3% | 5.0% | 4.7% | 5.7% | 6.9% |
|  |  | TAVI | 7.7% | 2.9% | 5.1% | 5.9% | 8.8% |
| **Breast** | Female | SAVR | 3.2% | 1.3% | 2.1% | 3.4% | 6.5% |
|  |  | TAVI | 4.0% | 1.7% | 2.3% | 2.0% | 9.9% |
| **CRC** | Male | SAVR | 4.6% | 1.6% | 2.9% | 4.8% | 6.1% |
|  |  | TAVI | 4.2% | 0.7% | 3.5% | 5.6% | 11.5% |
|  | Female | SAVR | 1.2% | 0.8% | 0.4% | 9.1% | 7.3% |
|  |  | TAVI | 4.8% | 0.8% | 4.0% | 3.2% | 9.5% |
| **Kidney** | Male | SAVR | 5.6% | 3.7% | 1.9% | 5.7% | 1.7% |
|  |  | TAVI | 6.1% | 0.0% | 5.9% | 0.0% | 11.9% |
|  | Female | SAVR | 0.0% | 0.0% | 0.0% | 7.0% | 4.9% |
|  |  | TAVI | 4.3% | 0.0% | 4.3% | 0.0% | 4.3% |
| **Lung** | Male | SAVR | 3.8% | 0.1% | 3.8% | 5.2% | 10.4% |
|  |  | TAVI | 2.9% | 0.01% | 2.9% | 10.3% | 2.9% |
|  | Female | SAVR | 7.4% | 1.0% | 6.4% | 0.0% | 8.2% |
|  |  | TAVI | 4.3% | 0.1% | 4.3% | 6.4% | 10.6% |
| **Prostate** | Male | SAVR | 4.0% | 1.3% | 2.9% | 3.5% | 5.8% |
|  |  | TAVI | 3.2% | 0.9% | 2.4% | 3.0% | 11.0% |
| **Age** |  |  |  |  |  |  |  |
| **No cancer** | Age<65 | SAVR | 4.8% | 2.4% | 2.6% | 4.8% | 4.8% |
|  |  | TAVI | 4.3% | 2.2% | 2.3% | 4.3% | 7.2% |
|  | Age>65 | SAVR | 8.8% | 4.5% | 4.8% | 6.3% | 6.8% |
|  |  | TAVI | 7.6% | 2.8% | 5.1% | 6.5% | 9.5% |
| **Breast** | Age<65 | SAVR | 1.3% | 0.0% | 1.3% | 2.5% | 2.4% |
|  |  | TAVI | 0.01% | 0.0% | 0.0% | 1.9% | 9.3% |
|  | Age>65 | SAVR | 3.5% | 1.3% | 2.3% | 3.4% | 6.7% |
|  |  | TAVI | 4.4% | 1.8% | 2.6% | 2.2% | 10.1% |
| **CRC** | Age<65 | SAVR | 3.3% | 0.0% | 3.3% | 0.0% | 3.3% |
|  |  | TAVI | 0.01% | 0.0% | 0.01% | 0.0% | 5.6% |
|  | Age>65 | SAVR | 3.2% | 1.4% | 1.8% | 7.0% | 6.8% |
|  |  | TAVI | 4.8% | 0.8% | 4.0% | 4.8% | 11.0% |
| **Kidney** | Age<65 | SAVR | 0.0% | 0.0% | 0.0% | 13.5% | 0.0% |
|  |  | TAVI | 0.0% | 0.0% | 0.0% | 0.0% | 16.7% |
|  | Age>65 | SAVR | 5.1% | 3.3% | 1.7% | 4.2% | 3.2% |
|  |  | TAVI | 6.6% | 0.0% | 6.5% | 0.0% | 8.1% |
| **Lung** | Age<65 | SAVR | 5.2% | 4.8% | 0.0% | 0.0% | 0.0% |
|  |  | TAVI | 0.0% | 0.01% | 0.0% | 6.2% | 0.0% |
|  | Age>65 | SAVR | 5.3% | 0.1% | 5.3% | 3.3% | 10.4% |
|  |  | TAVI | 4.0% | 0.1% | 4.0% | 9.1% | 7.1% |
| **Prostate** | Age<65 | SAVR | 0.0% | 0.0% | 0.0% | 2.8% | 5.6% |
|  |  | TAVI | 0.0% | 0.0% | 0.0% | 0.0% | 14.3% |
|  | Age>65 | SAVR | 4.2% | 1.4% | 2.9% | 3.5% | 5.8% |
|  |  | TAVI | 3.4% | 0.9% | 2.5% | 3.1% | 10.9% |

CRC: colorectal cancer, SAVR: surgical aortic valve replacement, TAVI: transcatheter valve implantation, AKI: acute kidney injury, PPM: permanent pacemaker.

**Supplemental Table 18: Propensity matched estimates between TAVI and SAVR among different age and gender in no cancer and all other cancers**

|  | **MACE** | **Mortality** | **Stroke** | **Major bleed** | **PPM** |
| --- | --- | --- | --- | --- | --- |
| **No cancer** |  |  |  |  |  |
| **Age ≤65** | 0.90(0.81-0.99) | 0.94(0.83-0.99) | 0.90(0.79-1.02) | 0.89(0.81-0.97) | 1.55(1.42-1.68) |
| **Age > 65** | 0.84(0.82-0.86) | 0.61(0.58-0.63) | 1.07(1.04-1.11) | 1.03(1.01-1.06) | 1.45(1.41-1.49) |
| **Male** | 0.81(0.79-0.85) | 0.70(0.66-0.74) | 1.03(0.99-1.08) | 1.01(0.97-1.05) | 1.61(1.56-1.69) |
| **Female** | 0.77(0.67-0.81) | 0.63 (0.61-0.66) | 0.78 (0.74-0.79) | 1.19 (0.50-1.61) | 1.4 (1.45-1.52) |
| **Prostate cancer** | | | | | |
| **Age ≤65** | - | - | - | - | 2.78(1.32-5.87) |
| **Age > 65** | 0.80(0.66-0.96) | 0.65(0.46-0.92) | 0.83(0.66-1, 03) | 0.85(0.70-1.03) | 2.02(1.77-2.31) |
| **Male** | 0.79(0.66-0.96) | 0.65(0.46-0.91) | 0.82(0.66-1.03) | 0.85(0.7-1.03) | 2.02(1.77-2.31) |
| **Lung cancer** | | | | | |
| **Age ≤65** | - | - | - | - | - |
| **Age > 65** | 0.75(0.50-1.14) | - | 0.75(0.50-1.14) | 2.95(1.97-4.43) | 0.66(0.48-0.90) |
| **Male** | 0.77(0.43-1.40) | - | 0.77(0.43-1.40) | 2.12(1.38-3.24) | 0.26(0.16-0.43) |
| **Female** | 0.56(0.32-0.98) | - | 0.66(0.37-1.17) | - | 1.33(0.86-2.06) |
| **Kidney Cancer** | | | | | |
| **Age > 65** | 1.31(0.8-2.13) | - | 4(1.98-8.08) | - | 2.64(1.54-4.53) |
| **Male** | 1.08(0.64-1.82) | - | 3.29(1.59-6.80) | - | 7.82(3.84-15.94) |
| **Female** | - | - | - | - | 0.86(0.35-2.12) |
| **Colorectal Cancer** | | | | | |
| **Age ≤65** | - | - | - | - | 1.74(0.58-5.21) |
| **Age > 65** | 1.54(1.15-2.06) | 0.58(0.34-1.02) | 2.27(1.59-3.24) | 0.66(0.52-0.84) | 1.68(1.38-2.05) |
| **Male** | 0.91(0.64-1.30) | 0.42(0.20-1.0) | 1.19(0.79-1.80) | 1.16(0.84-1.61) | 2.01(1.54-2.62) |
| **Female** | 3.98(2.21-7.16) | 1.00(0.40-2.46) | 9.29(3.69 -23.3) | 0.33(0.23-0.48) | 1.33(0.99-1.78) |
| **Breast Cancer** | | | | | |
| **Age ≤65** | - | - | - | 0.71(0.29-1.71) | 4.24(2.06-8.73) |
| **Age > 65** | 1.27(1.04-1.54) | 1.37(1.01-1.85) | 1.11(0.87-1.41) | 0.63(0.50-0.79) | 1.55(1.35-1.78) |
| **Female** | 1.25(1.03-1.52) | 1.33(0.98-1.79) | 1.11(0.87-1.41) | 0.58(0.46-0.73) | 1.57(1.37-1.79) |

AKI: acute kidney injury, PPM: permanent pacemaker.

**Supplemental Table 19: Main effect and Interaction analysis of the major outcomes based on the type of intervention and gender across patients with colorectal cancer**

| **Source** | **Dependent Variable** | **Type III Sum of Squares** | **Mean Square** | **F** | **Sig.** |
| --- | --- | --- | --- | --- | --- |
| **Intervention (TAVI vs. SAVR) * FEMALE** | MACE | 0.48 | 0.48 | 12.96 | <0.0001 |
|  | In-Hospital Mortality | 0.02 | 0.02 | 2.27 | 0.13 |
|  | Stroke | 0.29 | 0.29 | 10.68 | <0.0001 |
|  | Major Bleed | 1.38 | 1.38 | 26.86 | <0.0001 |
|  | PPM | 0.33 | 0.33 | 4.24 | 0.04 |

MACE: Major Adverse Cardiovascular Events; PPM: Permanent Pacemaker; AKI: acute kidney injury, SAVR: surgical aortic valve replacement, TAVI: transcatheter valve implantation,

**Supplemental Table 20: Main effect and Interaction analysis of the major outcomes based on the type of intervention and age across patients with colorectal cancer**

| **Source** | **Dependent Variable** | **Type III Sum of Squares** | **Mean Square** | **F** | **Sig.** |
| --- | --- | --- | --- | --- | --- |
| **Intervention (TAVI vs. SAVR) * agecat** | MACE | 0.182 | 0.182 | 4.951 | 0.026 |
|  | In-Hospital Mortality | 0.003 | 0.003 | 0.278 | 0.598 |
|  | Stroke | 0.231 | 0.231 | 8.485 | 0.004 |
|  | Major Bleed | 0.034 | 0.034 | 0.667 | 0.414 |
|  | PPM | 0.026 | 0.026 | 0.33 | 0.566 |

MACE: Major Adverse Cardiovascular Events; PPM: Permanent Pacemaker; AKI: acute kidney injury, SAVR: surgical aortic valve replacement, TAVI: transcatheter valve implantation

**Supplemental Table 21: Main effect and Interaction analysis of the major outcomes based on the type of intervention and gender across patients with lung cancer**

| **Source** | **Dependent Variable** | **Type III Sum of Squares** | **Mean Square** | **F** | **Sig.** |
| --- | --- | --- | --- | --- | --- |
| **Intervention (TAVI vs. SAVR) * FEMALE** | MACE | 0.09 | 0.09 | 2.14 | 0.14 |
|  | In-Hospital Mortality | 0.02 | 0.02 | 7.17 | 0.01 |
|  | Stroke | 0.03 | 0.03 | 0.76 | 0.38 |
|  | Major Bleed | 0.02 | 0.02 | 0.38 | 0.54 |
|  | PPM | 1.44 | 1.44 | 20.29 | <0.0001 |

MACE: Major Adverse Cardiovascular Events; PPM: Permanent Pacemaker; AKI: acute kidney injury, SAVR: surgical aortic valve replacement, TAVI: transcatheter valve implantation

**Supplemental Table 22: Main effect and Interaction analysis of the major outcomes based on the type of intervention and age across patients with lung cancer**

| **Source** | **Dependent Variable** | **Type III Sum of Squares** | **Mean Square** | **F** | **Sig.** |
| --- | --- | --- | --- | --- | --- |
| **Intervention (TAVI vs. SAVR) * agecat** | MACE | 0.08 | 0.08 | 1.902 | 0.168 |
|  | In-Hospital Mortality | 0.146 | 0.146 | 70.076 | <0.0001 |
|  | Stroke | 0.01 | 0.01 | 0.242 | 0.623 |
|  | Major Bleed | 0.001 | 0.001 | 0.018 | 0.893 |
|  | PPM | 0.059 | 0.059 | 0.831 | 0.362 |

MACE: Major Adverse Cardiovascular Events; PPM: Permanent Pacemaker; AKI: acute kidney injury, SAVR: surgical aortic valve replacement, TAVI: transcatheter valve implantation

**‘**

**Supplemental Table 23: Main effect and Interaction analysis of the major outcomes based on the type of intervention and gender across patients with renal cancer**

| **Source** | **Dependent Variable** | **Type III Sum of Squares** | **Mean Square** | **F** | **Sig.** |
| --- | --- | --- | --- | --- | --- |
| **Intervention (TAVI vs. SAVR) * FEMALE** | MACE | 0.11 | 0.11 | 2.47 | 0.12 |
|  | In-Hospital Mortality | 0.11 | 0.11 | 8.18 | <0.0001 |
|  | Stroke | 0 | 0 | 0 | 0.97 |
|  | Major Bleed | 0.01 | 0.01 | 0.21 | 0.65 |
|  | PPM | 0.91 | 0.91 | 16.32 | <0.0001 |

MACE: Major Adverse Cardiovascular Events; PPM: Permanent Pacemaker; AKI: acute kidney injury, SAVR: surgical aortic valve replacement, TAVI: transcatheter valve implantation

**Supplemental Table 24: Main effect and Interaction analysis of the major outcomes based on the type of intervention and age across patients with renal cancer**

| **Source** | **Dependent Variable** | **Type III Sum of Squares** | **Mean Square** | **F** | **Sig.** |
| --- | --- | --- | --- | --- | --- |
| **Intervention (TAVI vs. SAVR) * agecat** | MACE | 0.012 | 0.012 | 0.262 | 0.609 |
|  | In-Hospital Mortality | 0.062 | 0.062 | 4.668 | 0.031 |
|  | Stroke | 0.128 | 0.128 | 3.95 | 0.047 |
|  | Major Bleed | 0.475 | 0.475 | 16.511 | <0.0001 |
|  | PPM | 0.757 | 0.757 | 13.516 | <0.0001 |

MACE: Major Adverse Cardiovascular Events; PPM: Permanent Pacemaker; AKI: acute kidney injury, SAVR: surgical aortic valve replacement, TAVI: transcatheter valve implantation

**Supplemental Table 25: Main effect and Interaction analysis of the major outcomes based on the type of intervention and age across patients with breast cancer**

| **Source** | **Dependent Variable** | **Type III Sum of Squares** | **Mean Square** | **F** | **Sig.** |
| --- | --- | --- | --- | --- | --- |
| **Intervention (TAVI vs. SAVR) * agecat** | MACE | 0.102 | 0.102 | 2.914 | 0.041 |
|  | In-Hospital Mortality | 0.005 | 0.005 | 0.367 | 0.045 |
|  | Stroke | 0.049 | 0.049 | 2.195 | 0.139 |
|  | Major Bleed | 0.002 | 0.002 | 0.073 | 0.788 |
|  | PPM | 0.263 | 0.263 | 3.499 | 0.061 |

MACE: Major Adverse Cardiovascular Events; PPM: Permanent Pacemaker; AKI: acute kidney injury, SAVR: surgical aortic valve replacement, TAVI: transcatheter valve implantation

**Supplemental Table 26: Main effect and Interaction analysis of the major outcomes based on the type of intervention and age across patients with prostate cancer**

| **Source** | **Dependent Variable** | **Type III Sum of Squares** | **Mean Square** | **F** | **Sig.** |
| --- | --- | --- | --- | --- | --- |
| **Intervention (TAVI vs. SAVR) * agecat** | MACE | 0.006 | 0.006 | 0.173 | 0.678 |
|  | In-Hospital Mortality | 0.002 | 0.002 | 0.194 | 0.66 |
|  | Stroke | 0.002 | 0.002 | 0.09 | 0.764 |
|  | Major Bleed | 0.05 | 0.05 | 1.601 | 0.206 |
|  | PPM | 0.121 | 0.121 | 1.585 | 0.208 |

MACE: Major Adverse Cardiovascular Events; PPM: Permanent Pacemaker; AKI: acute kidney injury, SAVR: surgical aortic valve replacement, TAVI: transcatheter valve implantation

**Supplemental Figure 1: Propensity matched variables and degree of covariate balances across the comparison groups**


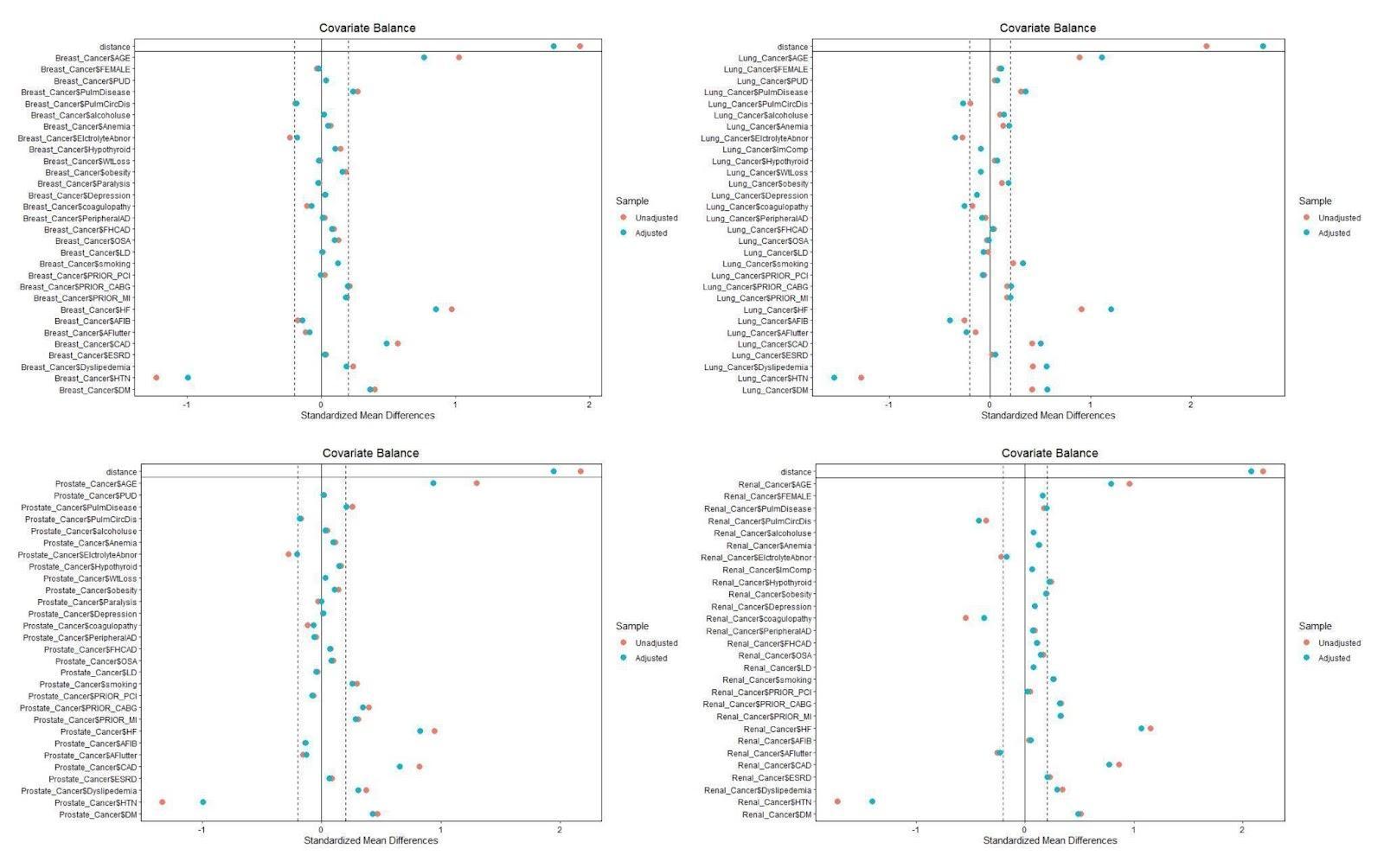


**Supplemental Figure 2: Absolute standardized differences in means, distribution of propensity scores and kernel density estimation of the all and propensity matched populations**


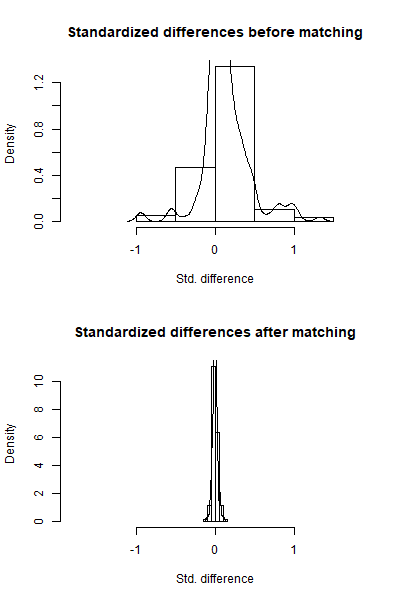

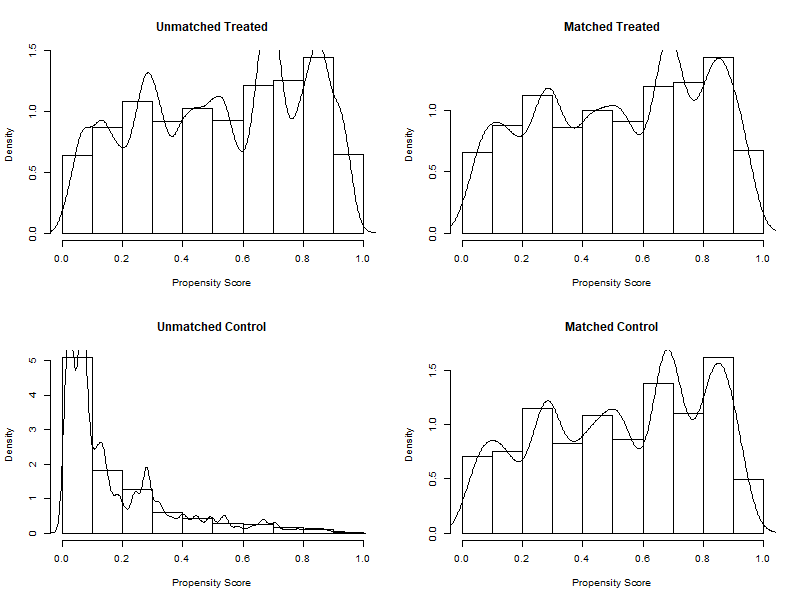


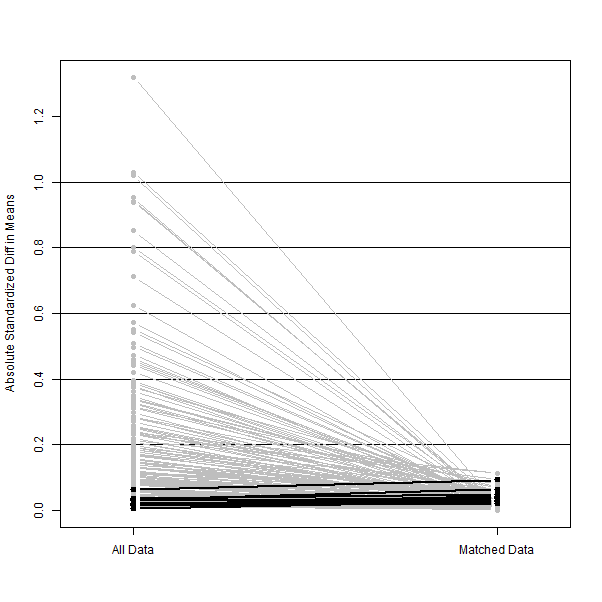

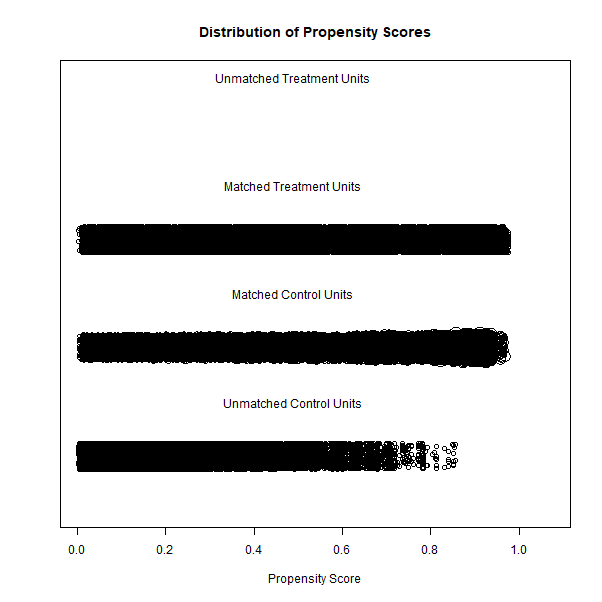


**Supplemental Figure 3: Flow chart demonstrating the algorithm for population selection for propensity-matched analysis**


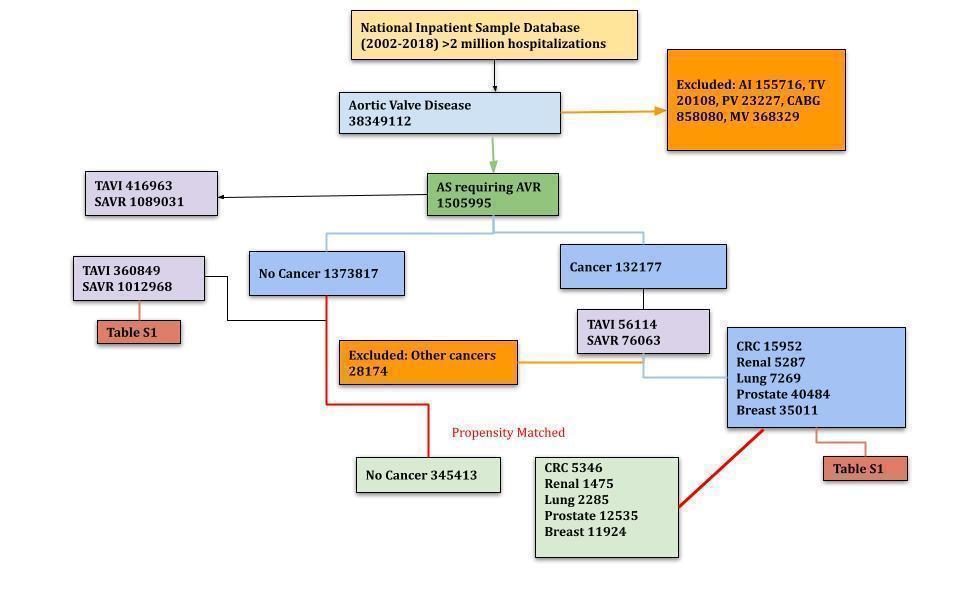


CRC: colorectal cancer, TAVI: transcatheter valve implantation, SAVR: surgical aortic valve replacement

**Supplemental Figure 4: Baseline comorbidities of patients with all type cancers undergoing TAVI vs. SAVR**


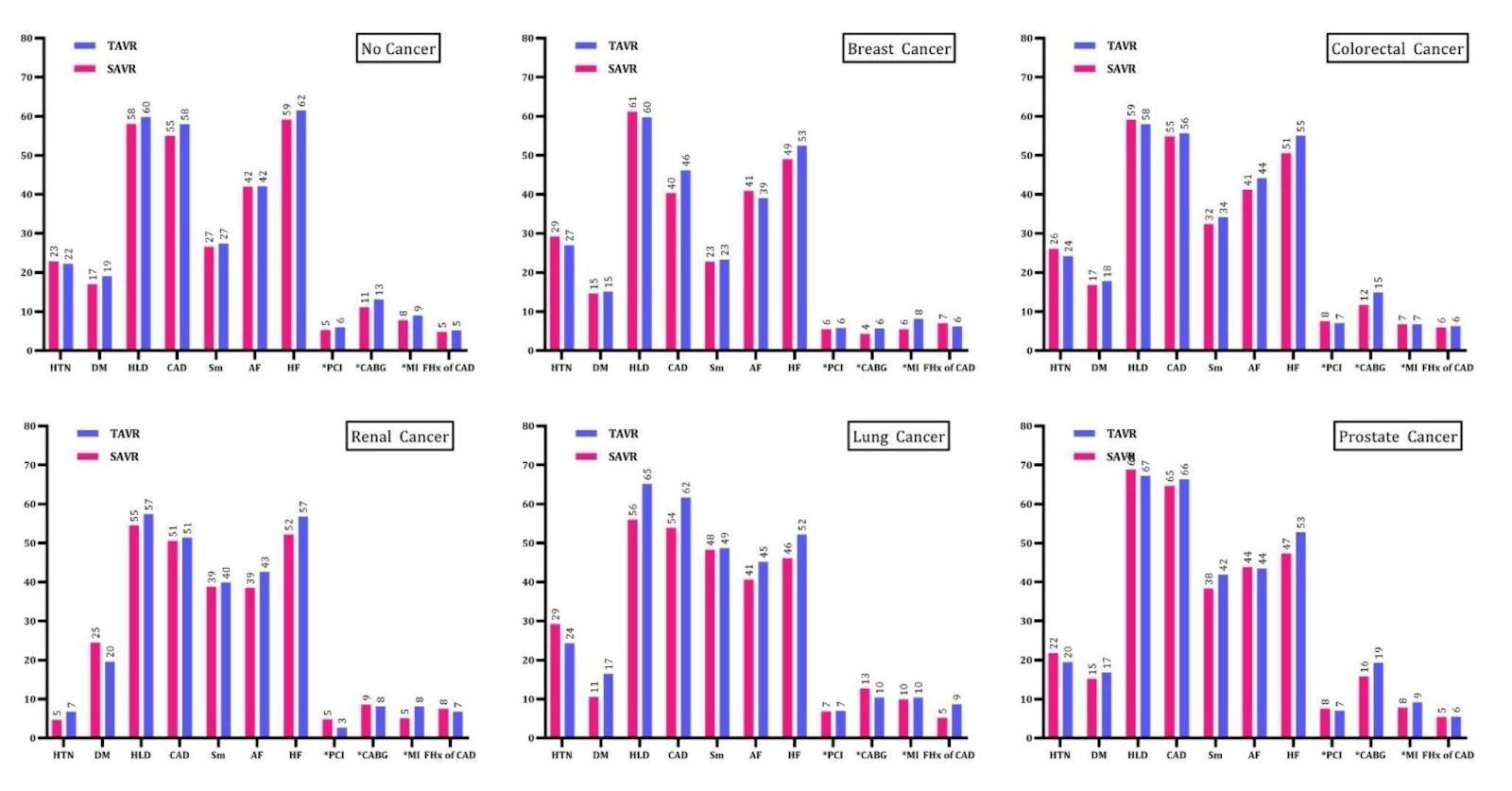


TAVI: transcatheter valve implantation, SAVR: surgical aortic valve replacement

**Supplemental Figure 5: Yearly prevalence of AS and trend of SAVR and TAVI performed on patients with no cancer and all cancers.**

1. Yearly prevalence of patients with AS in no-cancer and cancer patients


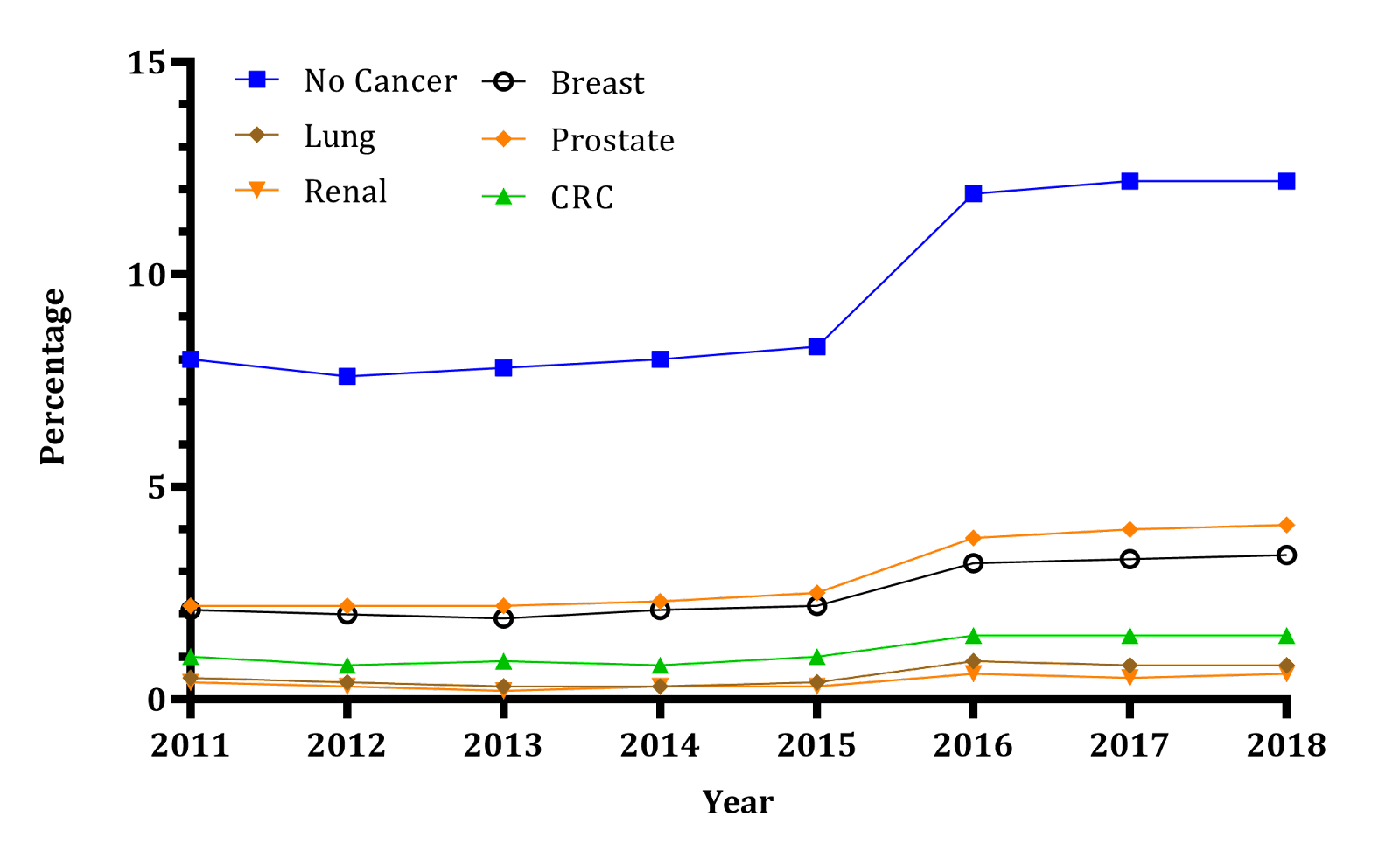


1. Yearly trend of SAVR vs. TAVI


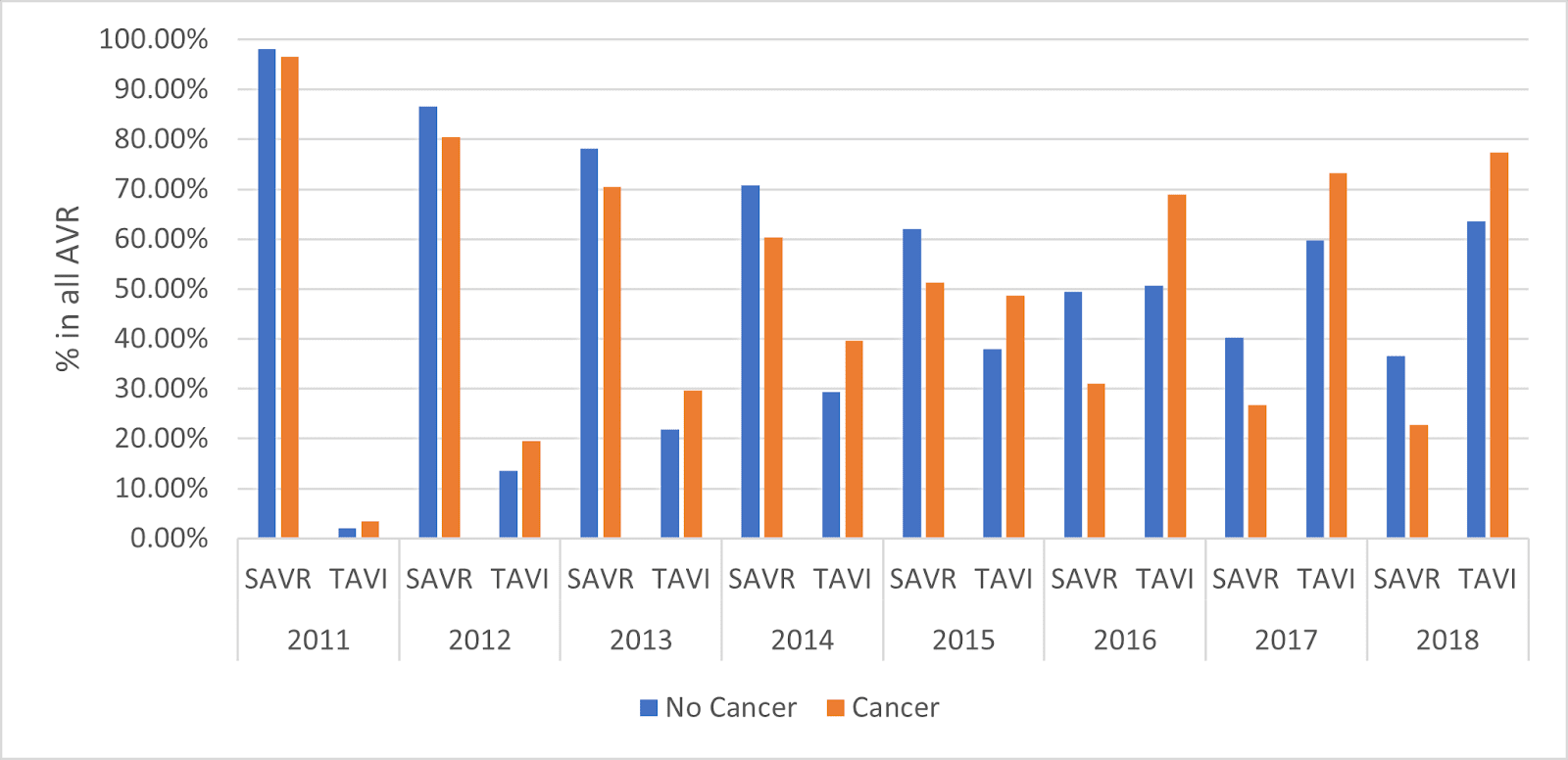


**Supplemental Figure 6: A bivariate analysis of components of MACE (death and stroke) for propensity-matched estimates of different cancer patients undergoing SAVR vs. TAVI**


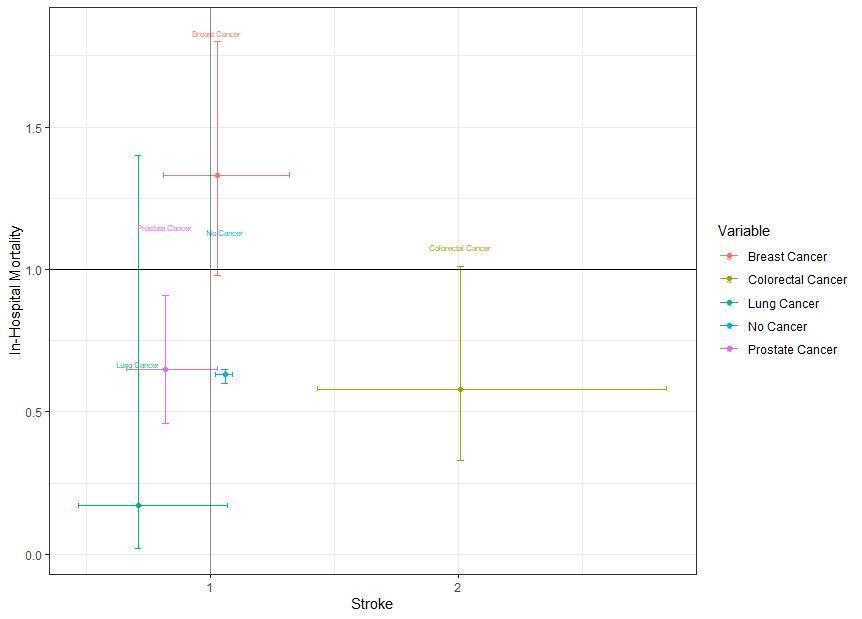


**Supplemental Figure 7: Length of Stay (LOS) in days among patients with different cancers**


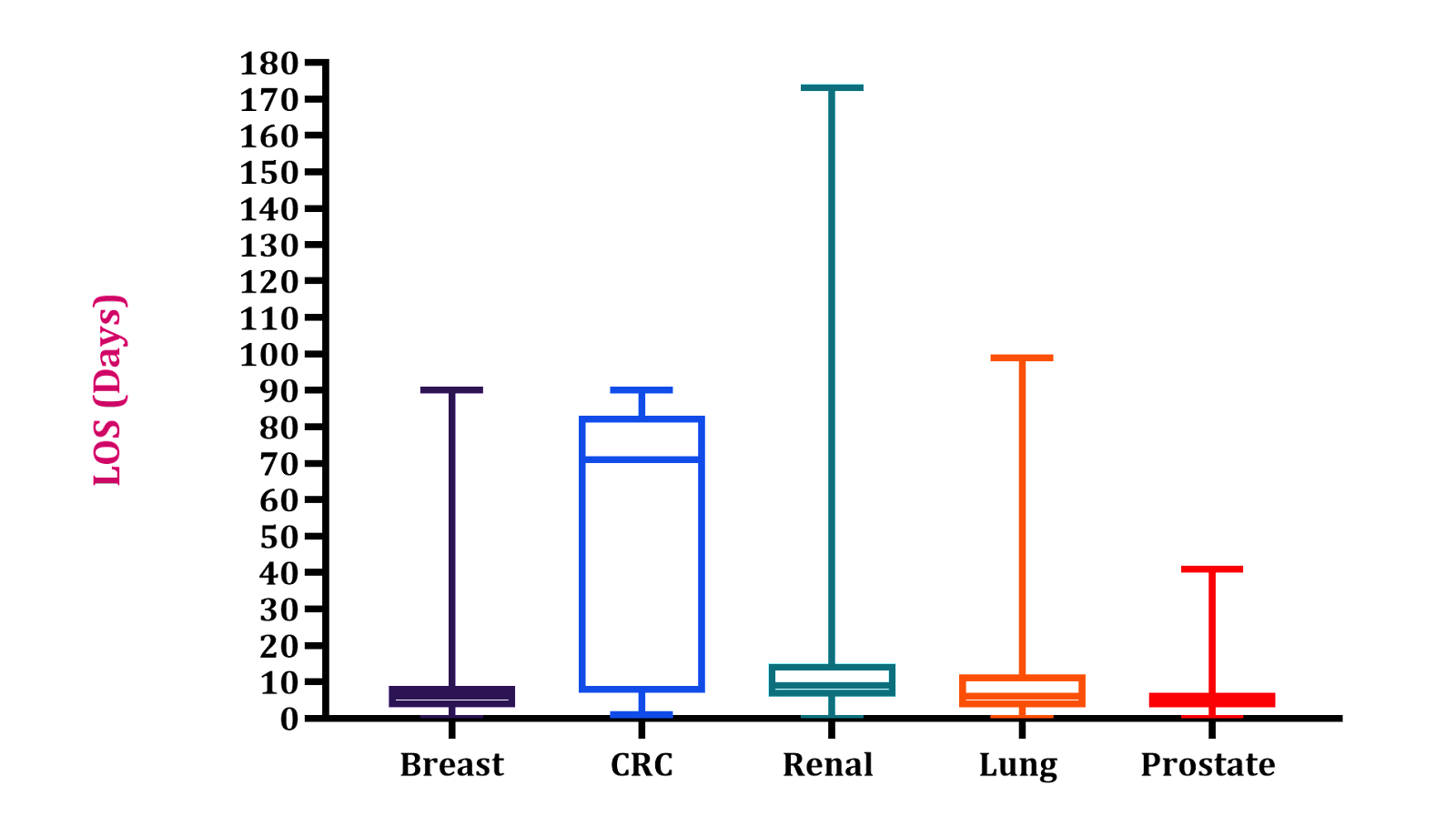


CRC: colorectal cancer

**Supplemental Figure 8: Interaction analysis of age with type of intervention (TAVI and SAVR) for patients with breast cancer**


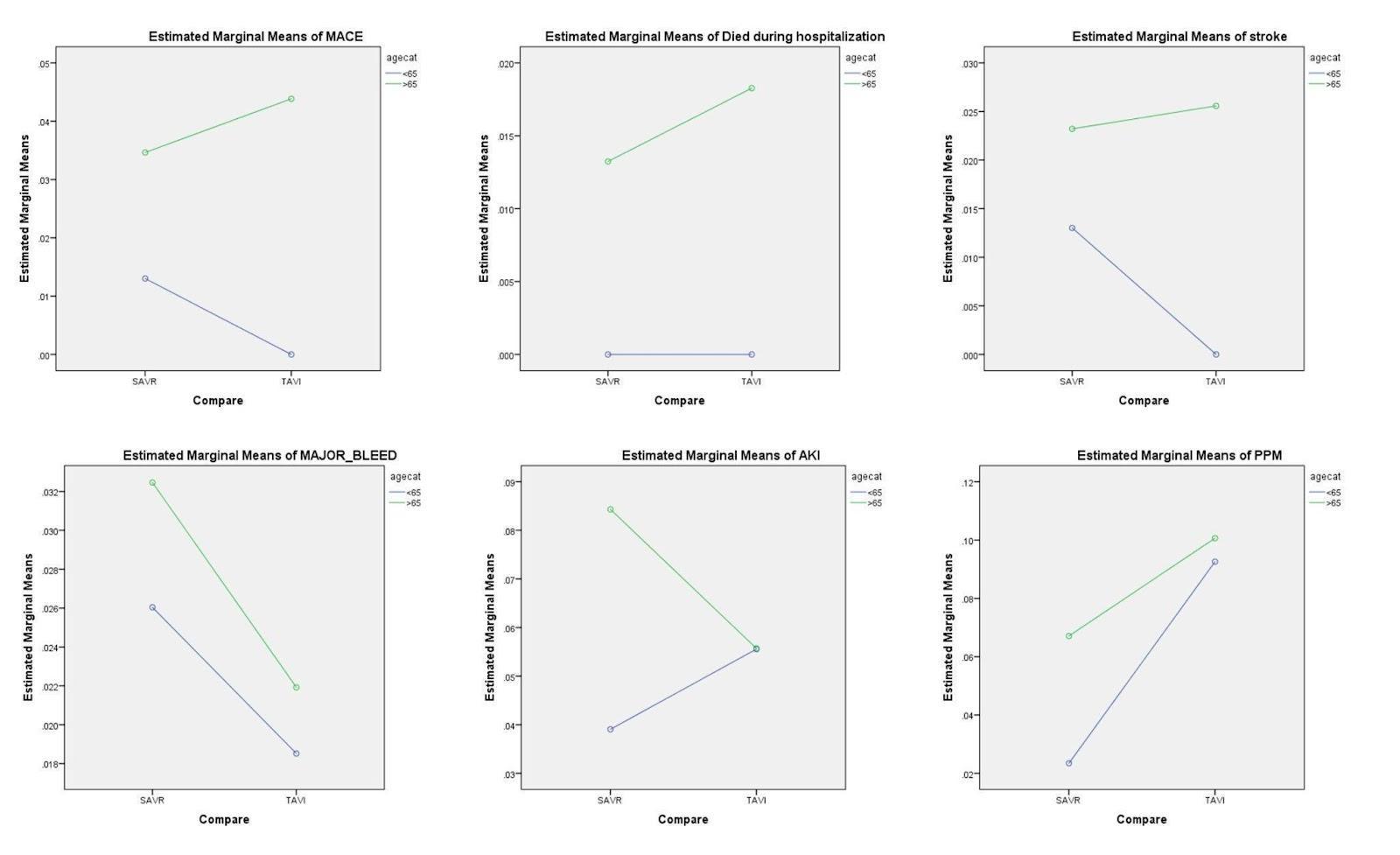


TAVI: transcatheter valve implantation, SAVR: surgical aortic valve replacement, MACE: major adverse cardiovascular events, PPM: permanent pacemaker, AKI: acute kidney injury

**Supplemental Figure 9: Interaction analysis of age with type of intervention (TAVI and SAVR) for patients with prostate cancer**
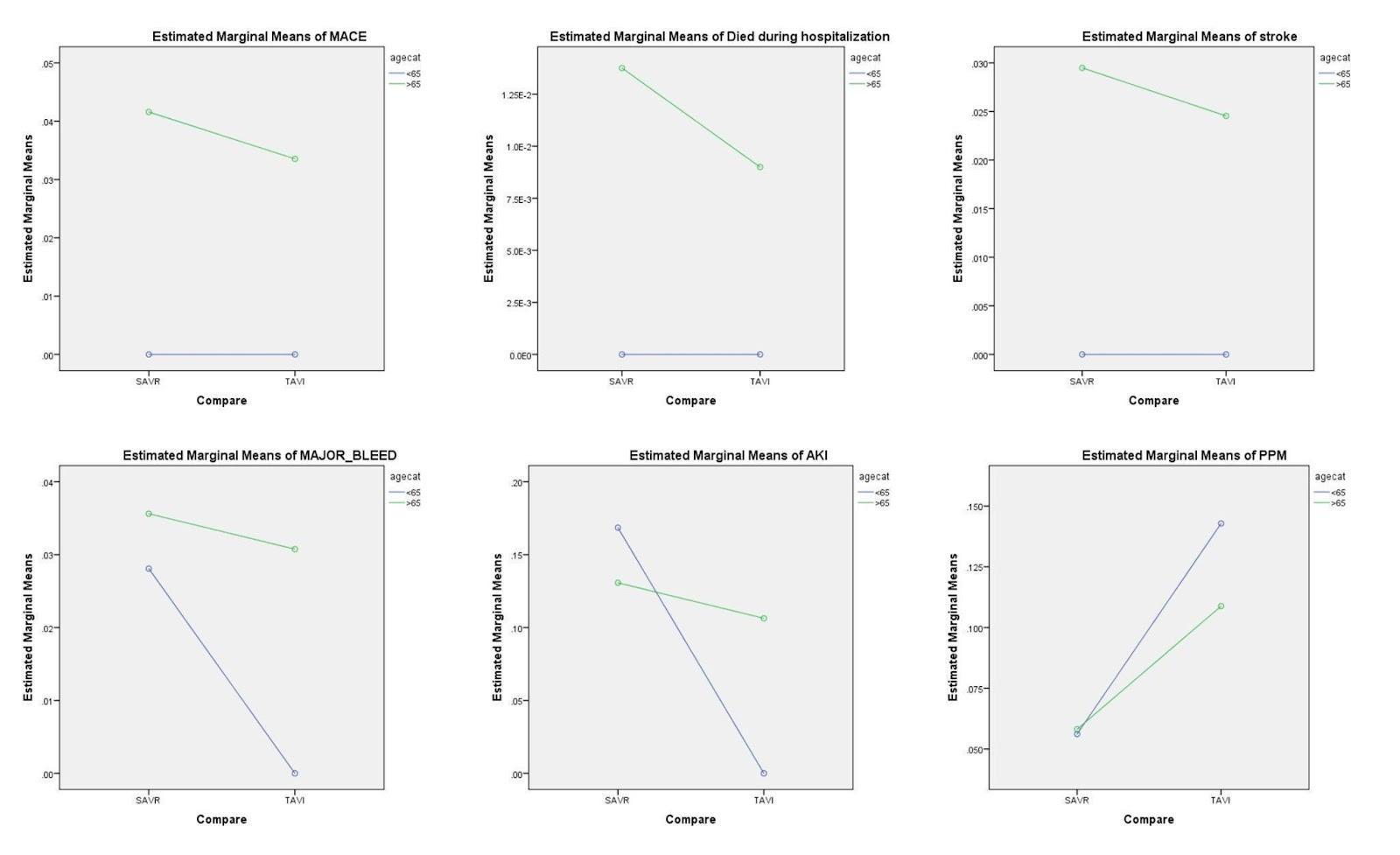


TAVI: transcatheter valve implantation, SAVR: surgical aortic valve replacement, MACE: major adverse cardiovascular events, PPM: permanent pacemaker, AKI: acute kidney injury

**Supplemental Figure 10: Interaction analysis of age with type of intervention (TAVI and SAVR) for patients with colorectal cancer**
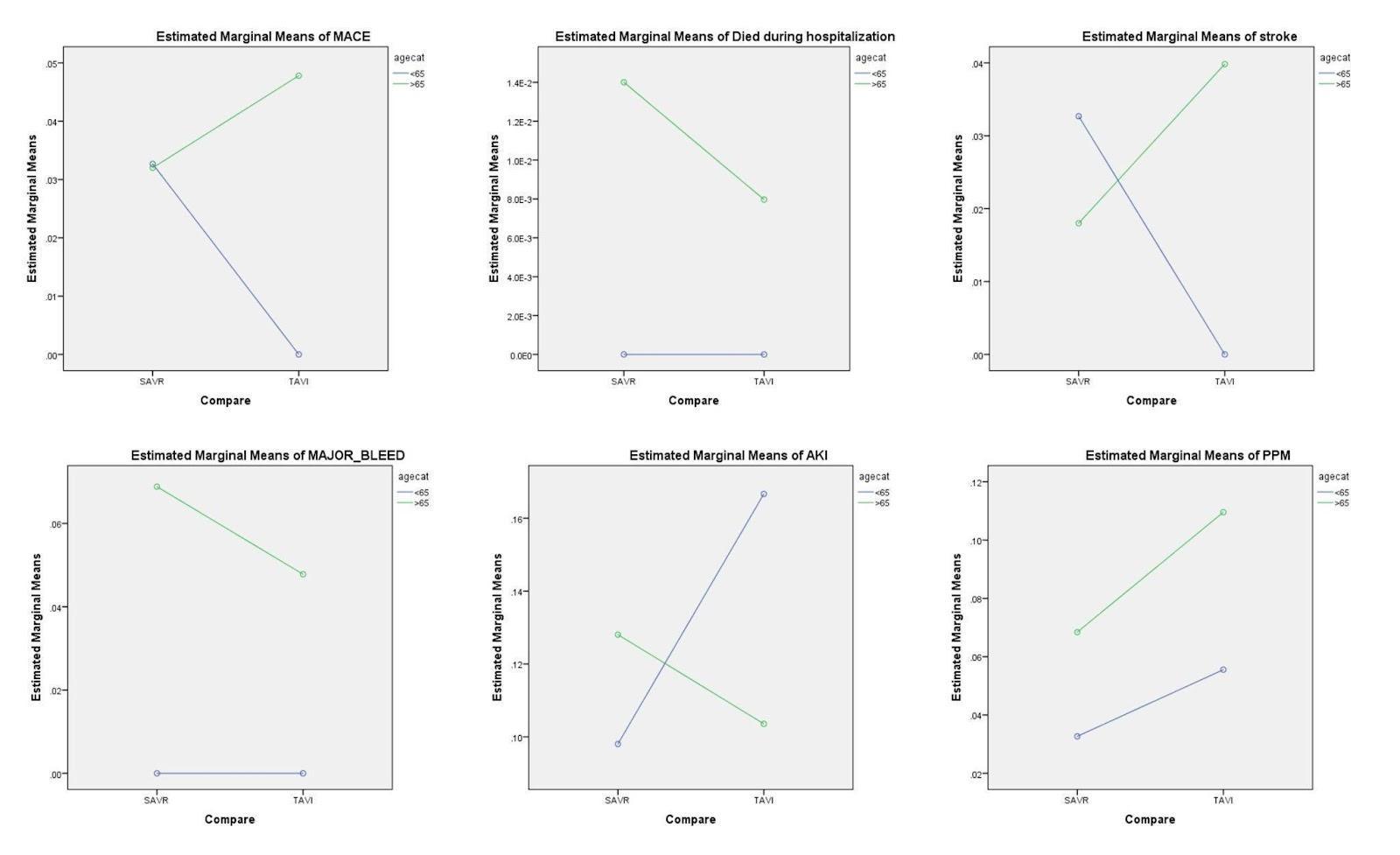


TAVI: transcatheter valve implantation, SAVR: surgical aortic valve replacement, MACE: major adverse cardiovascular events, PPM: permanent pacemaker, AKI: acute kidney injury

**Supplemental Figure 11: Interaction analysis of gender with type of intervention (TAVI and SAVR) for patients with colorectal cancer**
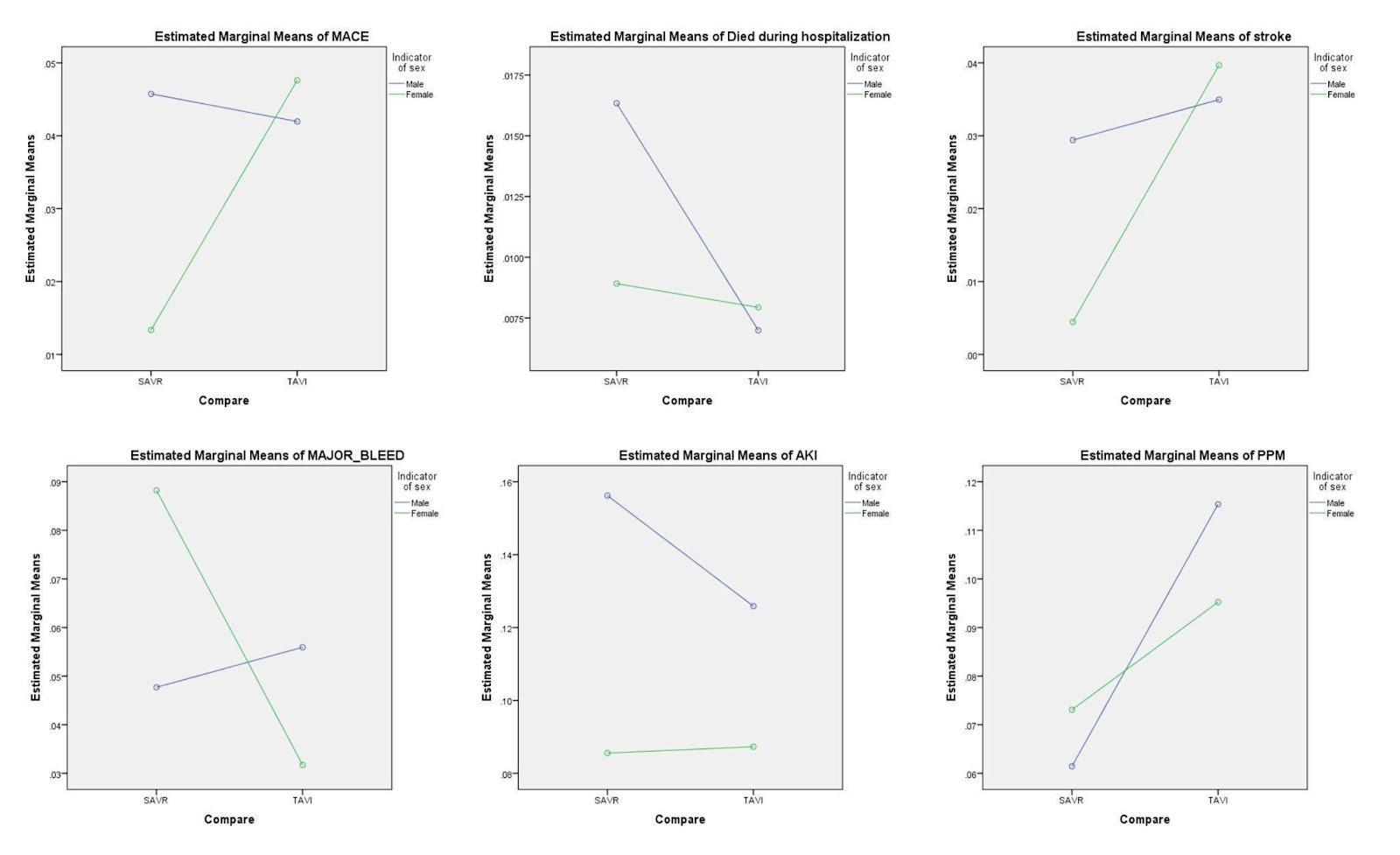


TAVI: transcatheter valve implantation, SAVR: surgical aortic valve replacement, MACE: major adverse cardiovascular events, PPM: permanent pacemaker, AKI: acute kidney injury

**Supplemental Figure 12: Interaction analysis of age with type of intervention (TAVI and SAVR) for patients with lung cancer**


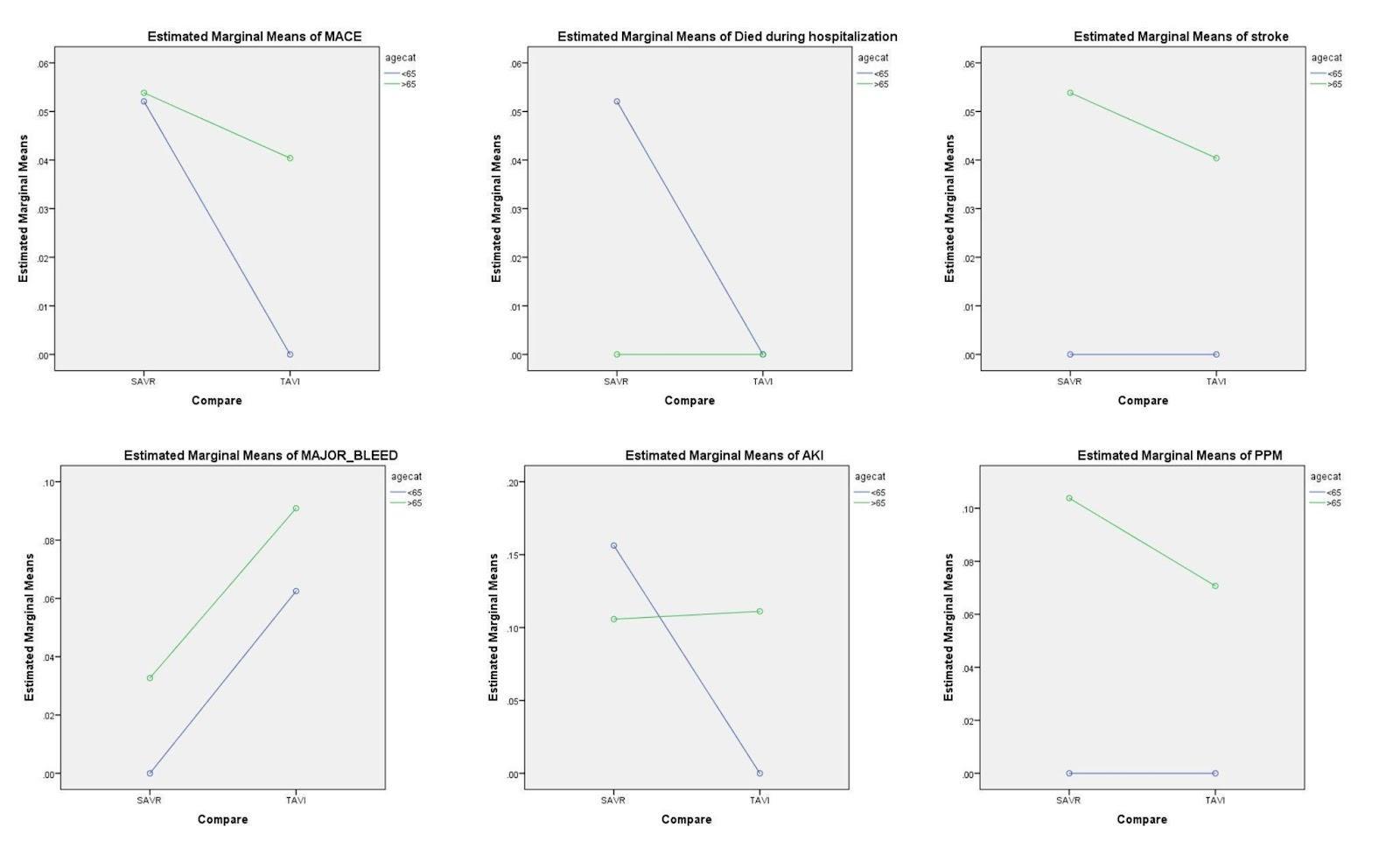


TAVI: transcatheter valve implantation, SAVR: surgical aortic valve replacement, MACE: major adverse cardiovascular events, PPM: permanent pacemaker, AKI: acute kidney injury

**Supplemental Figure 13: Interaction analysis of gender with type of intervention (TAVI and SAVR) for patients with lung cancer**
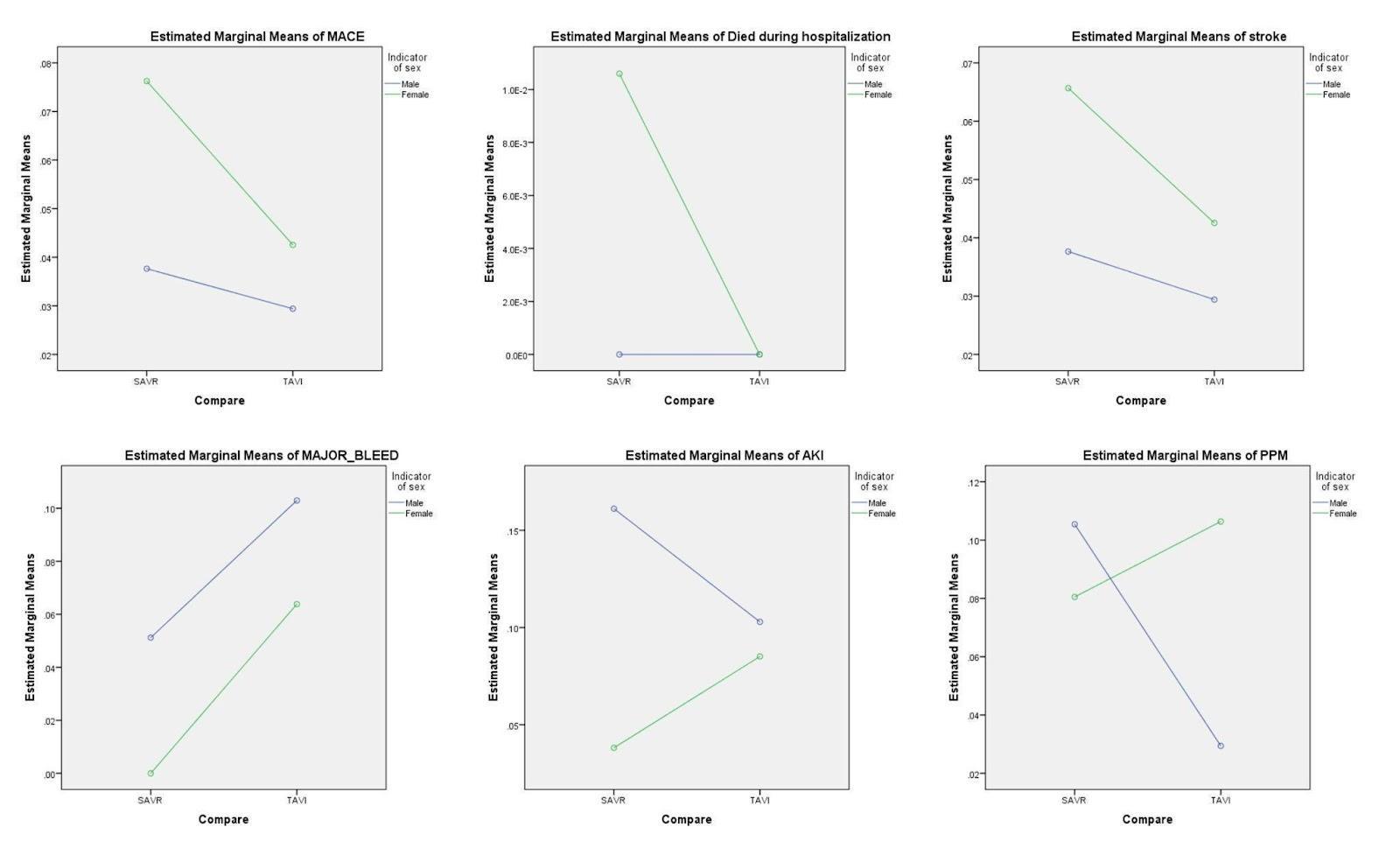


TAVI: transcatheter valve implantation, SAVR: surgical aortic valve replacement, MACE: major adverse cardiovascular events, PPM: permanent pacemaker, AKI: acute kidney injury

**Supplemental Figure 14: Interaction analysis of age with type of intervention (TAVI and SAVR) for patients with** **renal cancer**


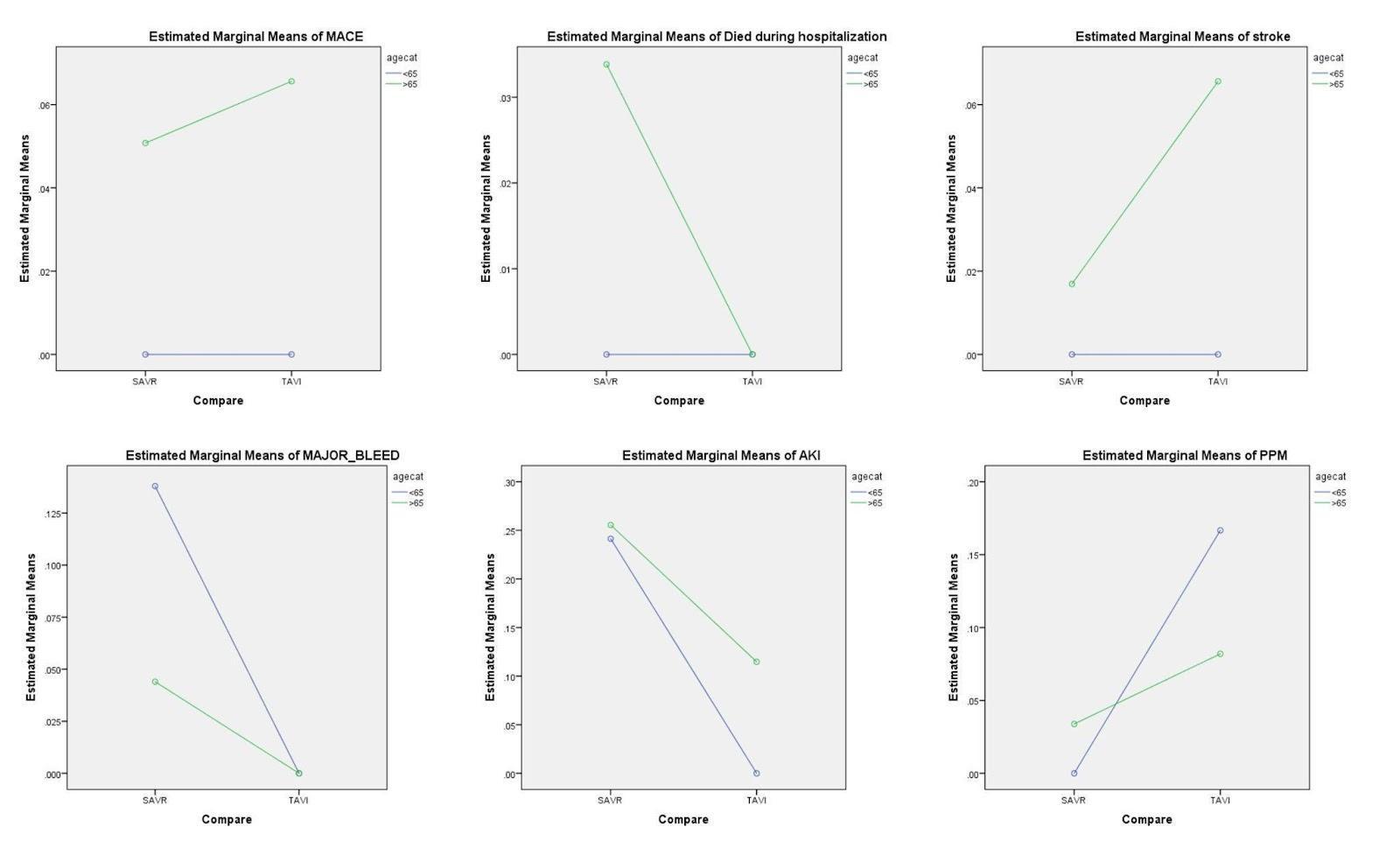


TAVI: transcatheter valve implantation, SAVR: surgical aortic valve replacement, MACE: major adverse cardiovascular events, PPM: permanent pacemaker, AKI: acute kidney injury

**Supplemental Figure 15: Interaction analysis of gender with type of intervention (TAVI and SAVR) for patients with renal cancer**
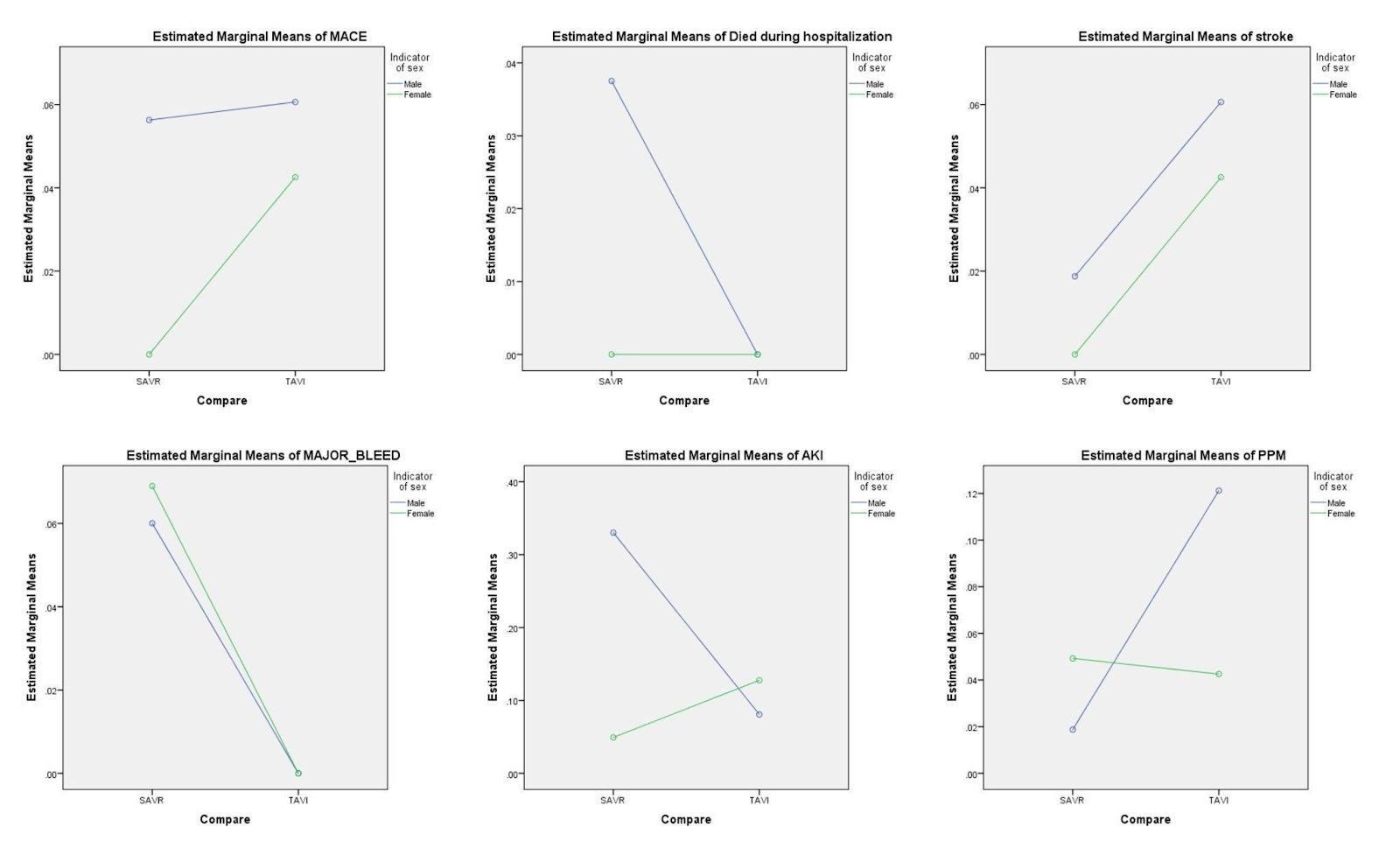


TAVI: transcatheter valve implantation, SAVR: surgical aortic valve replacement, MACE: major adverse cardiovascular events, PPM: permanent pacemaker, AKI: acute kidney injury
